# Marine cloud brightening

**DOI:** 10.1098/rsta.2012.0086

**Published:** 2012-09-13

**Authors:** John Latham, Keith Bower, Tom Choularton, Hugh Coe, Paul Connolly, Gary Cooper, Tim Craft, Jack Foster, Alan Gadian, Lee Galbraith, Hector Iacovides, David Johnston, Brian Launder, Brian Leslie, John Meyer, Armand Neukermans, Bob Ormond, Ben Parkes, Phillip Rasch, John Rush, Stephen Salter, Tom Stevenson, Hailong Wang, Qin Wang, Rob Wood

**Affiliations:** 1 National Centre for Atmospheric Research, Boulder, CO 80301, USA; 2 Department of Atmospheric Sciences, University of Washington, Seattle, WA 98105, USA; 3 Climate Science, Pacific Northwest National Laboratory, Richland, WA 99352, USA; 4 School of Earth and Atmospheric Sciences, University of Manchester, Manchester M13 9PL; 5 MACE, University of Manchester, Manchester M13 9PL, UK; 6 NCAS, SEE, University of Leeds, Leeds LS2 9JT, UK; 7 Department of Engineering, University of Edinburgh, Edinburgh EH9 3JL, UK; 8 FICER, CA, USA

**Keywords:** cloud brightening, geoengineering, albedo, cloud modelling, spray technology, field experiment

## Abstract

The idea behind the marine cloud-brightening (MCB) geoengineering technique is that seeding marine stratocumulus clouds with copious quantities of roughly monodisperse sub-micrometre sea water particles might significantly enhance the cloud droplet number concentration, and thereby the cloud albedo and possibly longevity. This would produce a cooling, which general circulation model (GCM) computations suggest could—subject to satisfactory resolution of technical and scientific problems identified herein—have the capacity to balance global warming up to the carbon dioxide-doubling point. We describe herein an account of our recent research on a number of critical issues associated with MCB. This involves (i) GCM studies, which are our primary tools for evaluating globally the effectiveness of MCB, and assessing its climate impacts on rainfall amounts and distribution, and also polar sea-ice cover and thickness; (ii) high-resolution modelling of the effects of seeding on marine stratocumulus, which are required to understand the complex array of interacting processes involved in cloud brightening; (iii) microphysical modelling sensitivity studies, examining the influence of seeding amount, seed-particle salt-mass, air-mass characteristics, updraught speed and other parameters on cloud–albedo change; (iv) sea water spray-production techniques; (v) computational fluid dynamics studies of possible large-scale periodicities in Flettner rotors; and (vi) the planning of a three-stage limited-area field research experiment, with the primary objectives of technology testing and determining to what extent, if any, cloud albedo might be enhanced by seeding marine stratocumulus clouds on a spatial scale of around 100×100 km. We stress that there would be no justification for deployment of MCB unless it was clearly established that no significant adverse consequences would result. There would also need to be an international agreement firmly in favour of such action.

## 1. Introduction

Marine cloud brightening (MCB), one of several solar radiation management (SRM) geoengineering ideas involving the production of a global cooling to compensate for the warming associated with continuing fossil fuel burning, was first postulated by Latham [1,2]. The ideas, engineering requirements and some climate impacts associated with MCB have been significantly explored by more recent studies [3–11].

The basic principle behind the idea is to seed marine stratocumulus clouds with sea water aerosol generated at or near the ocean surface. These particles would have sufficiently large salt mass to ensure their activation and subsequent growth within the clouds, without being so large as to encourage precipitation formation. Moreover, they would be sufficiently numerous to enhance the cloud droplet number concentration (CDNC) to values substantially higher than the natural ones, thereby enhancing the cloud albedo [12]. Increasing the CDNC is likely also to affect macrophysical properties such as cloud cover, longevity, liquid water content and thickness, as a consequence of inhibiting precipitation formation [13], and the time scale for the evaporation and sedimentation of cloud droplets. These feedbacks on the cloud properties can result in secondary aerosol indirect effects that are poorly understood and represent a major challenge in the general problem of understanding and quantifying how aerosols impact the climate system [14,15].

General circulation model (GCM) simulations suggest that, if the droplet number concentration in marine stratocumulus could be increased to several hundred per cubic centimetre in a significant fraction of the stratocumulus sheets, then—subject to satisfactory resolution of various problems mentioned later—a negative forcing could be produced, sufficient to balance approximately the warming associated with carbon dioxide doubling, and maintain the polar sea-ice coverage at roughly current values. However, the computations of Rasch *et al.* [6] indicated that the negative forcing required to hold the Earth’s average surface temperature at the current value would be different from that required for average sea-ice coverage maintenance (which would in fact be different at the two poles). Latham *et al.* [5] outlined observational studies that give some support for the viability of MCB, but it cannot be regarded as definitive.

Current major problems regarding MCB, which may or may not be capable of resolution, are

— we do not yet have a spraying system capable of producing sea water particles of the size and in the copious quantities required;
— even if we succeeded in producing such a system, we would still need to ensure that it would function satisfactorily at sea for long periods (we envisage several months) in the face of problems such as bad weather, possible orifice clogging, etc.;
— we need to ascertain whether we could produce sea water cloud-condensation nuclei (CCN) at a sufficient rate, over a wide enough area, for enough of them to enter the marine stratocumulus clouds and be activated to produce cloud droplets, thereby enhancing the CDNC *N* and the associated cloud albedo *A* sufficiently to produce the required degree of cooling (the work of Korhonen *et al.* [9] and Wang *et al.* [16]—and others—illustrates how the cloud and sub-cloud characteristics are much more complex than assumed in our GCM modelling); and
— if the earlier mentioned problems were satisfactorily resolved, and a limited-area field investigation of MCB demonstrated its quantitative viability, there would be no case for its deployment unless (i) comprehensive examination demonstrated that there would be no unacceptable ramifications and (ii) a not yet established international body, representing all countries, concluded—after major investigation of all evidence available—that deployment was needed and safe.

This is not a conventional study. It is essentially a description and assessment of ‘work in progress’, with an accompanying look ahead to our future studies. It focuses attention on all elements of the research we (the authors of this study) have conducted since the publication of our three papers, Salter *et al.* [4], Latham *et al.* [5]—which constituted a review of all work on MCB up to that point—and Rasch *et al.* [6], a fully coupled GCM study that concentrated on the possibility of maintaining or restoring, via MCB, global average surface temperature, rainfall and polar sea-ice coverage, to roughly current values. For reasons of space, we do not reproduce herein, except cursorily, results from those studies: we simply refer to them. The content of this study embraces both scientific and technological work and covers about six separate topics. It is therefore difficult to provide a fully comprehensive analysis of each individual component of our overall research programme—or, indeed, of papers by other authors, on or related to MCB.

A well-recognized crucial question pertaining to all SRM techniques concerns the unintended, possibly deleterious, consequences that might result from their deployment, which should never occur before full international approval is granted (as mentioned earlier), and a fully comprehensive assessment of all ramifications of deployment have been openly published and debated. A full discussion and analysis of all possible socio-political impacts of deployment of MCB would be far too lengthy to be incorporated into this study, and should, in any event, be undertaken by experts in that important area, which we are not, and so we confine ourselves—except in §6, concerned with field testing of MCB—to underlining the critical need for such an assessment to be made, and making brief references to it at appropriate points. This study is essentially restricted to the science and technology of our work on MCB.

One issue that affects, directly or indirectly, virtually all of the separate components of our MCB research programme concerns the size and number concentration of the droplets naturally occurring in all marine stratocumulus clouds that might be candidates for seeding, in all seasons and in all locations over the globe.

**Figure 1.**
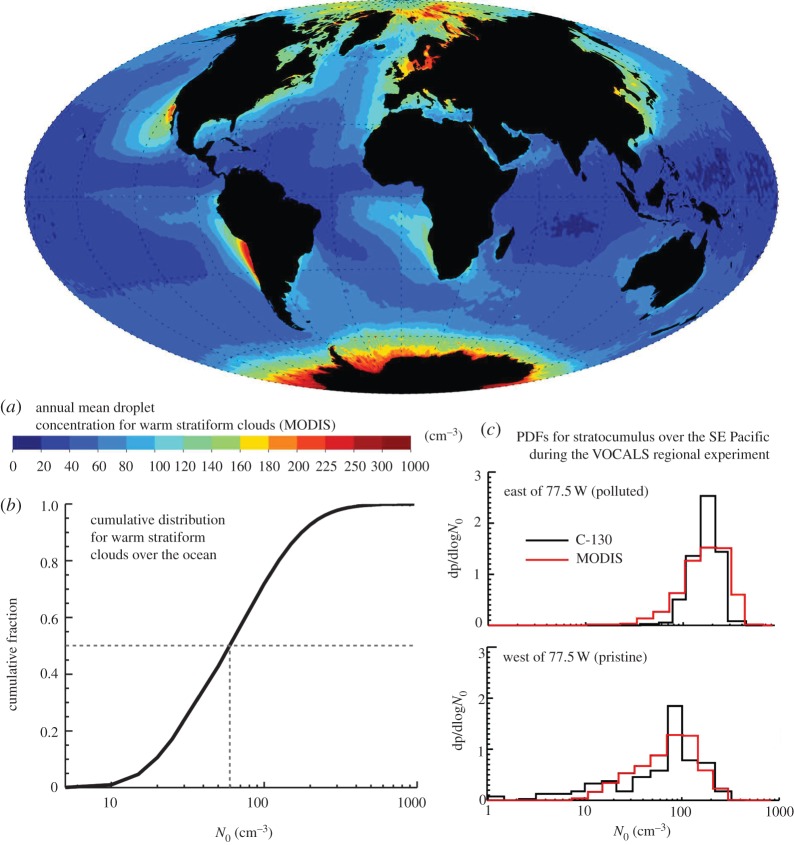
(*a*) Map of MODIS-derived annual mean cloud droplet concentration, *N*_0_, for stratiform marine warm clouds. To be included in the annual mean, the daily warm cloud fraction in 1×1^°^ boxes must exceed 50% to capture primarily marine stratocumulus clouds. (*b*) Cumulative distribution of daily 1×1^°^ droplet number, *N*_0_, from MODIS for all ocean points. (*c*) A comparison of MODIS and C-130 aircraft-measured cloud droplet concentration estimates from the VOCALS regional experiment during October/November 2008 off the Chilean coast [18], for longitudes 70–77.5^°^ W (more polluted) and 77.5–85^°^ W (more pristine). There is good agreement between *in situ* and satellite-derived values that lends weight to the use of these data over the global oceans.

The change in cloud albedo resulting from seeding the clouds with sea water particles large enough to be activated is roughly proportional to 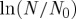 [12], where *N*_0_ and *N* are, respectively, the background droplet number concentration (prior to seeding) and the post-seeding value. *N*_0_ is therefore a critical parameter in determining the albedo enhancement resulting from seeding; so it is crucial to obtain accurate values of *N*_0_, over the oceans. Recent observational work [17,18], based on data from the NASA MODIS satellite instrument and airborne measurements in the VOCALS field experiment, are beginning to provide more reliable global distributions of *N*_0_ values than have been available hitherto. These findings are illustrated in figure 1. The preferred regions for seeding are those with lower values of *N*_0_, which will change with the seasons. More detailed descriptions of the choice of seeding regions are presented in our earlier papers [4–6].

This study is organized as follows: (i) introduction; (ii) GCM modelling of MCB, with emphasis on rainfall and sea-ice amounts and distributions; (iii) high-resolution cloud modelling; (iv) parcel modelling and its application to our spray technology; (v) spray-production techniques and modelling of Flettner rotor instabilities by computational fluid dynamics (CFD); (vi) planning of a limited-area field research experiment to test MCB and enhance our fundamental knowledge of marine stratocumulus clouds; and (vii) discussion.

A rough outline description of the linkages between these somewhat disparate sections is as follows. The GCM computations (§2) provide estimates of the changes made by prescribed cloud seeding to the values and global distributions of salient parameters such as cloud albedo, top of atmosphere (TOA) forcing, surface temperature (detailed in Latham *et al.* [5]; so not duplicated herein), rainfall, sea-ice cover (see Rasch *et al.* [6]) and sea-ice thickness. These studies are all based on a much-simplified picture of cloud properties, and do not take account of the complexities of the upward transport into cloud base of some fraction of the sea water aerosol, generated close to the ocean surface. The high-resolution cloud modelling (§3) follows the work of Korhonen *et al.* [9] and Wang *et al.* [16], which take much more detailed account of these complexities. The parcel modelling (§4) examines the sensitivity of cloud–albedo change to the numbers and salt masses *m*_*s*_ of sea water aerosol entering the clouds, as a function of values of *N*_0_, updraught speed and other cloud parameters. This work provides the estimates of the ranges of sea water droplet size that are required of the spray system, i.e. values that will produce droplets of salt masses *m*_*s*_ sufficient to be activated on entry to the clouds, but small enough not to promote unwanted drizzle development. The current stages of the development of two types of spray system (electrohydrodynamic spray fabrication and micro-fabrication lithography) are described comprehensively in §5 and in Salter *et al.* [4], respectively. This earlier work also presents, in detail, the further development of (and case for) utilization of unmanned, wind-powered Flettner-rotor vessels as vehicles from which the sea water particles could be sprayed. Section 5 of this study gives an account of a CFD study of Flettner rotors, designed to help optimize their performance. Section 6 presents an outline of a three-stage, limited-area field research experiment that may be performed at some future point if approved (as discussed earlier) and if vindicated by information available after completion of the work described in this paragraph, as well as by the research of others. The geoengineering objective of the field experiment would be to conduct a quantitative study—for a variety of situations—of the extent to which maritime clouds can be made more reflective by seeding them with sea water aerosol. The field experiment, probably conducted on a spatial scale of about 100×100 km, is not designed to examine any associated climate changes. Section 7 presents a discussion of the recent work on MCB described in earlier sections, and attempts to define the research questions most in need of early resolution.

## 2. Global climate modelling: precipitation and ice cover

This section describes research conducted using the UK Met Office climate model—the Hadley Centre global environmental model (HadGEM1)—to study some climatological impacts of changing the CCN concentration in defined maritime oceanic regions that have significant stratocumulus sheets. We present the studies of the influence of this seeding on global precipitation and polar sea-ice extent and thickness. In §2*a*, changes in precipitation resulting from MCB seeding are discussed; in §2*b*, new results are presented on the MCB impacts on ice thickness and ice extent.

The HadGEM1 model used in our current studies is based on version 6.1 of the UK Met Office’s unified model (UM), with an atmospheric resolution of 1.25×1.875^°^ with 38 vertical levels, an upper lid at 39 km and a coupled ocean model of variable grid size from 1^°^ squares at the poles to one-third of a degree at the Equator and to a depth of 5.3 km, using 40 levels. An emphasis in these models is on the improvement in the stratocumulus cloud mixing parametrizations, and this has been particularly useful in MCB studies, enabling improved calculations to be made of cloud droplet effective radius, radiative forcing and liquid water path [19]. They have also provided the ability to focus on precipitation, surface temperature, cloud and sea surface temperatures (SSTs), ice fraction and depth [20].

There have been several GCM studies of MCB since the first atmosphere-only simulations [5]. HadGAM, an atmosphere-only climate model, has the advantage of an immediate response to greenhouse gas forcing, and can provide an immediate change in the TOA radiative forcing. It is limited by having no component of ocean meridional heat flux and circulation. Slab GCMs have the advantage that short-time-scale thermocline changes are simulated. This can be suitable for numerical weather prediction purposes, but is of limited value in climate studies. Fully coupled ocean–atmosphere GCMs include the large-scale oceanic meridional heat transport, but the long-time-constant ocean circulations provide the challenge of large-scale hysteresis for the climate system. Climate models are typically used to simulate time scales of decades to centuries. It is necessary to allow for significant spin-up time, permitting slow response processes within the climate system to fully react to the new environment, with only the later, stable years used for analysis. Deep ocean circulations and sea-ice changes are examples of important long-time-coefficient processes. These same climate models are used to investigate the long-term effects of geoengineering scenarios.

Jones *et al.* [7,8] investigated the impacts of stratocumulus seeding over three regions using HadGAM and HadGEM models of the UK Met Office. They assumed that, following seeding, the CDNC (*n*) was maintained at *n*=375 cm^−3^ throughout the seeding regions. Bala *et al.* [10] and Rasch *et al.* [6] used the National Center for Atmospheric Research’s community climate system model. Rasch *et al.* [6] investigated the effects of seeding the most susceptible 20 per cent, 40 per cent and 70 per cent of marine stratocumulus clouds. This work sets *n*=1000 cm^−3^ and has a changing seeding pattern. Bala *et al.* [10] simulated seeding by reducing the effective radius of cloud droplets in all suitable marine clouds. Results from these four studies show a significant increase in albedo, equivalent to compensating for an approximate doubling of pre-industrial planetary atmospheric carbon dioxide. For the atmosphere-only HadGAM computations, the equivalent TOA negative forcing is about −3.7 W m^−2^.

Korhonen *et al.* [9] used the GLOMAP-bin model, which contains explicit aerosol microphysics in an offline transport mode to estimate the cloud drop number response to a wind-speed-dependent emission function. They found that if they used spray-droplet production rates similar to those estimated by earlier studies [2,4] the *n* values resulting from seeding were substantially less than 375 cm^−3^, with concomitant reduced values of negative forcing *F* being well below those emanating from the GCM studies cited earlier. Their study indicates that higher emission rates would be required to achieve substantial forcing, and that seeding could actually decrease CDNC in some regions. Another possible explanation for the disparity between the *F*-values obtained by other workers and Korhonen *et al.* [9] is that the values of ambient (pre-seeding) droplet concentration used in the latter study are appreciably higher than those used in the GCM computations that are based on the values shown in figure 1. Additionally, H. Korhonen (2011, personal communication) suggests that the vertical velocity field distribution used in their simulations could have been too small, and this may be the reason why their background (no seeding) stratocumulus cloud droplet concentrations (*N*_0_) are higher than the observations (figure 1) and the GCM fields.

Both classes of global model studies are gross simplifications of the real world. The former assumes that it is straightforward to change CDNC but allow a response in meteorological features (e.g. boundary layer stability, cloud cover, turbulence) and climate (e.g. surface temperature and precipitation). The latter treats aerosol cloud drop formation more accurately but neglects the meteorological and climate response. Each class of study provides useful but incomplete information about this geoengineering strategy.

In our study, three simulations were completed, each for 70 years from 2020 to 2090, with the last 20 years analysed: a control run with static carbon dioxide levels at 2020 levels (440 ppm); a continued global warming simulation (2CO_2_) based on the control run plus 1 per cent CO_2_ increase per annum until double the pre-industrial level is reached (560 ppm) in 2045 and thereafter held constant; the MCB case, which is the same as the preceding but with droplet number increased to *n*=375 cm^−3^ in three regions. These are off the western coasts of California, Peru and Namibia, which Jones *et al.* [7] highlighted as being particularly effective, owing to their propensity for stratocumulus cloud fields in our current climatology. These three regions were also seeded in GCM studies by Latham *et al.* [5].

### (a) Precipitation

There is no doubt that, if any SRM geoengineering technique were deployed, it would produce changes in rainfall patterns and amounts. A crucial question surrounding all SRM techniques is whether such deployment would produce a reduction in rainfall, in any cultivated regions, which would result in a significant reduction in agricultural yield. If so, this SRM technique should be abandoned, unless some safe way is found of modifying the technique or operational procedures to redress the situation in this same region.

There have been several published studies of the effect of MCB on global rainfall ([6–8,10], using the models outlined earlier). Further work, using the same model as Jones *et al.*, is described herein. In the important and influential paper by Jones *et al.* [7], the three-patch seeding procedure described earlier was used, with the imposed CDNC *n*=375 cm^−3^. Their most noteworthy finding was a significant reduction in precipitation for the whole-averaged Amazon basin. This finding has been confirmed in our recent studies. Rasch *et al.* [6], on the other hand, who seeded over significantly larger cloudy areas, ranging from 20 to 70 per cent of the total area covered by suitable clouds, found no reduction in rainfall in this region, whereas Bala *et al.* [10], who seeded all suitable clouds, found a smaller but discernible rainfall reduction over a small fraction of this Amazonian region. When Jones *et al.* [8] repeated their earlier studies, except that they did not seed the Southern Atlantic patch of stratocumulus cloud, they found that there was no reduction in rainfall in the Amazonian region.

There is no definitive understanding of the reasons for the variations in results described in the preceding paragraph. It seems likely, however, that the locations and relative amounts of seeding are important factors in governing the rainfall changes. If this proves to be true, then in principle, if MCB was ever safely capable of functioning in the manner assumed in our GCM computations (please note the various caveats regarding MCB made in §1 and in later parts of this study), there would be some latitude to vary the location of seeding in order—hopefully—to eliminate specified adverse effects. This possible flexibility would be highest in the early years or decades of a deployment programme, when the fraction of clouds seeded would be low.

The study of Bala *et al.* [10] indicates—again, subject to the earlier mentioned caveats—that MCB seeding sufficient to produce a global cooling that would roughly balance the warming resulting from carbon dioxide doubling would cause a globally averaged rainfall reduction of 1.3 per cent. However, this study also shows a global land-based moistening, with an average increase in precipitation over land of 3.5 per cent. Bala *et al.* attribute this enhancement of precipitation over land to the flow of moist air from ocean to land, created by the cooling resulting from cloud albedo enhancement.

**Figure 2.**
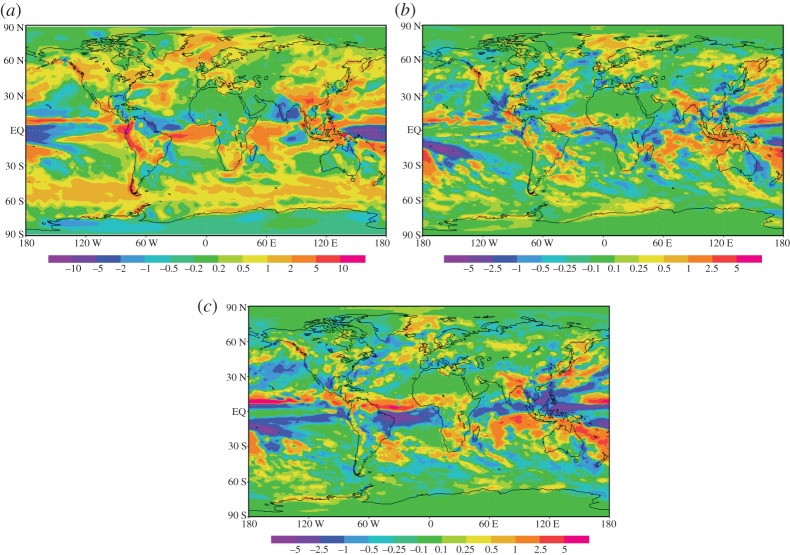
A comparison between model and observed precipitation, and investigation into the impacts of MCB on model precipitation (mm per day). (*a*) Comparison of the CMAP precipitation dataset with a current CO_2_ level simulation in HadGEM1. (*b*) The effect of increasing carbon dioxide from 440 to 560 ppm within the model. (*c*) The difference between a geoengineered simulation, 2CO_2_ and a control simulation. (Online version in colour.)

Precipitation is not well described in climate models. The Climate Prediction Center Merged Analysis of Precipitation (CMAP) dataset provided by the National Oceanic and Atmospheric Administration [21] for 1979–2000 was compared with a 10 year simulation using current static carbon dioxide levels. Figure 2*a* shows the difference between the precipitation rate in HadGEM1 and the CMAP dataset. The globally averaged difference in precipitation rates over land is an increase of 0.17 mm per day. The current globally averaged global precipitation for the control run (CON) minus the observations (CMAP) is 0.44 mm per day, corresponding to figure 2*a*. The global difference in precipitation for 2CO_2_–CON simulations is 0.0035 mm per day (figure 2*b*) and for the MCB–CON simulation is 0.0068 mm per day (figure 2*c*). Across most of the northern land masses, the precipitation difference is less than 1 mm per day. However, in some regions, this still results in a doubling of precipitation. In the tropical regions, the model does not reproduce well measured values downwind of particularly the Southeast Asian and South American mountain ranges; this may also be consistent with a small increase in precipitation in the stratocumulus regions in the southern hemisphere. Across the globe, the model is the weakest in the presence of steep mountain ranges, on the west of a continental region. The increased precipitation on the upwind steep slopes produces an impact on the availability of water vapour in the lee of the mountains, and this has been specifically discussed earlier for the Amazonian region.

Figure 2*b* shows the difference between the control case and the 2CO_2_ case. In the double carbon dioxide atmosphere, there appears to be an increase in precipitation over the Southeast Asian rainforests and the southern extent of Brazil, where there is an increase of less that 10 per cent of the original rainfall. Furthermore, India is subject to between 1 and 2.5 mm per day increase. However, this is closer to a 50 per cent increase in regional precipitation. Figure 2*c* is the comparison between the MCB and the CON simulations. Figure 2*c* is similar to fig. 4*b* in Jones *et al.* [7], fig. 3*b* in Rasch *et al.* [6] and fig. 7 in Bala *et al.* [10]. Although each model has used a different seeding strategy, there is some degree of overlap. The reduction of precipitation in figure 2*c* for the whole-averaged Amazon basin is consistent with that of Jones *et al.* [7,8]. This amounts to an over 50 per cent reduction in precipitation over the most easterly point of South America. Thus, our results and those of Jones *et al.* [8] should be treated with caution in this region. Excess precipitation on the upwind steep slopes of the Andes removes downwind available atmospheric water vapour. This reduction is not present in Rasch *et al.* [6], but they seed a much larger portion of the ocean. In the African subcontinent, our results produce a band of increased precipitation over the Sahel, and so, as already mentioned, we need to treat all these results with caution. African and Indonesian precipitation increases are also present in Rasch *et al.* [6].

**Figure 3.**
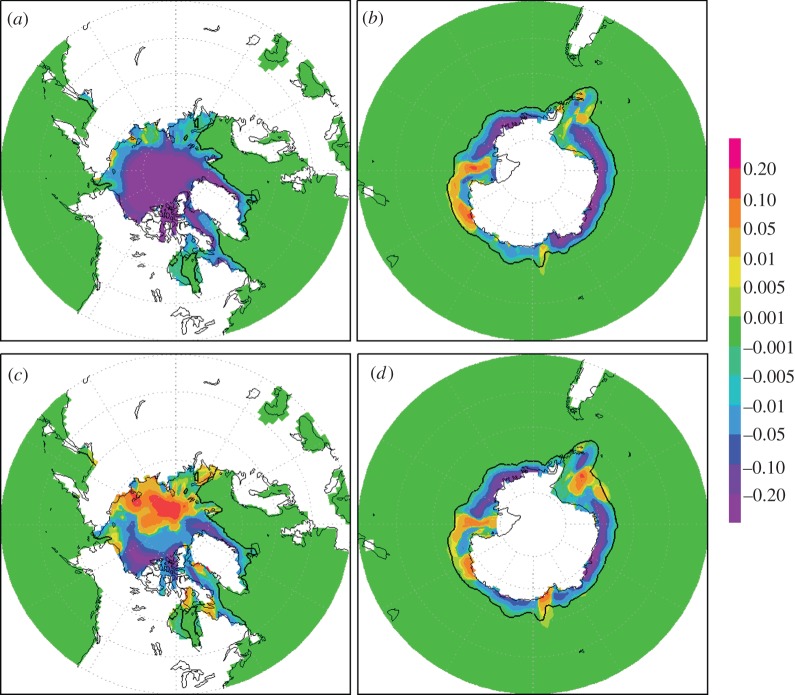
A comparison of the north and south polar sea-ice fraction averaged over the summer minimum for the final 20 years of the 70 year simulations. Sea-ice fraction can be interpreted as the fraction of time that ice is present at that location. The northern minimum is taken as September, and the southern minimum is taken as March. (*a*,*b*) The difference in north and south polar sea-ice fraction between 2CO_2_ and CON. (*c*,*d*) The difference in north and south polar sea-ice fraction between MCB and CON. The black contour shows the ice limit in CON. (Online version in colour.)

**Figure 4.**
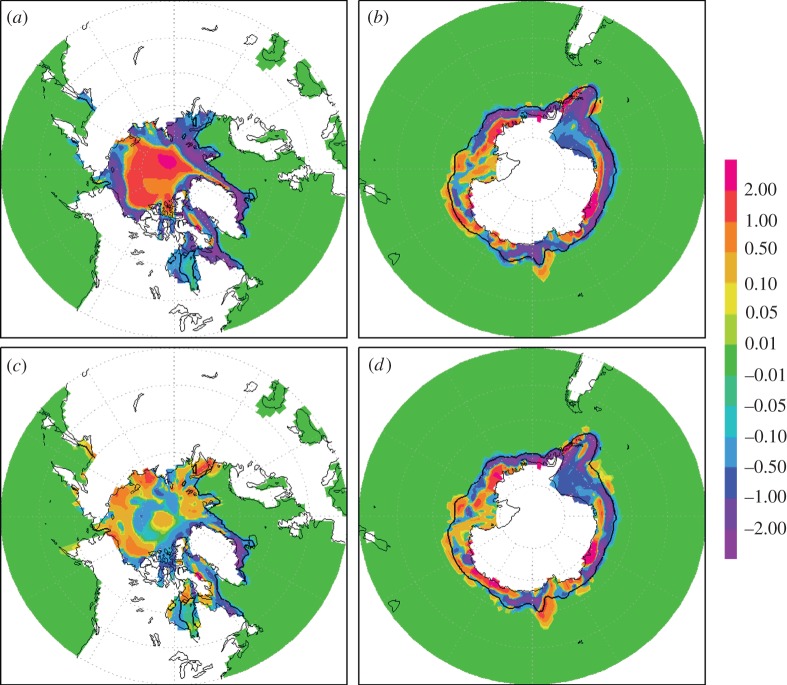
A comparison of the north and south polar sea-ice thickness (m) averaged over the summer minimum for the final 20 years of 70 year simulations. The northern minimum is taken as September, and the southern minimum is taken as March. (*a*,*b*) The difference in the north and south polar sea-ice thickness between 2CO_2_ and CON. (*c*,*d*) The difference in the north and south polar sea-ice thickness between MCB and CON. The black contour shows the ice limit in CON. (Online version in colour.)

To summarize, one of the most difficult challenges in climate modelling is to predict more accurately global precipitation patterns [20]. Our results contribute to this discussion. They show a small increase in precipitation in the drier regions of Africa, as indicated in figure 2*b*,*c*, with up to 5 mm per day average decrease in the Amazon region in the two scenarios of a 2CO_2_ and a MCB climate. These results from our model simulations indicate that there are changes in precipitation produced in the seeding cases, but that the variations are within the bounds of current model precision and uncertainties. Higher resolution and more accurate simulations are clearly required for future work on this.

### (b) Sea-ice extent and thickness

Figures 3 and 4 show the change in the summer minimum sea-ice fraction and sea-ice thickness, respectively. The Arctic ice minimum has been taken to occur in September and the Antarctic minimum in March. Similar to precipitation, sea ice is not well represented in climate models. Winston [22] argues that the ice cover is more sensitive than climate models suggest. Even though our results are therefore likely to be underestimated, they do show significant changes, and further analysis seems merited.

Figure 3*a*,*c* shows the difference between the 2CO_2_ and CON simulations and indicates a significant reduction in sea-ice fraction under a doubling of pre-industrial carbon dioxide atmosphere. There is a general and significant loss of sea ice in polar regions under double carbon dioxide levels. In the Southern Hemisphere (figure 3*b*,*d*), the reduction in sea ice is non-uniform, with the most significant reduction to be found east of the Antarctic Peninsula. The Arctic ice minimum in the double carbon dioxide scenario (figure 3*a*, 2CO_2_–CON) is a 76 per cent reduction from the 2020 ice extent, but with seeding switched on (figure 3*b*), MCB–CON, the reduction is only 3 per cent. In the Southern Hemisphere (figure 3*c*,*d*), the equivalent reductions are 30 per cent and 17 per cent, respectively. These relative changes in sea-ice fraction match the SST fields, where, in the Northern Hemisphere, 2CO_2_ increase over CON is 1.4 K, and the Southern Hemisphere results in an increase of 0.4 K. In the MCB case, these increases are reduced to −0.2 and 0.3 K, respectively.

In contrast to what has been stated earlier, the sea-ice depth increases close to the North Pole (figure 4*a*,*b*), creating a small central region of thicker ice in the 2CO_2_ scenario, and to a much lesser extent in the MCB scenario. This increase in sea-ice thickness in the 2CO_2_ case corresponds to an increase in north polar precipitation. In the Southern Ocean, the changes are non-uniform and, in some existing ice regions, there is an increase in the south polar minima sea-ice thickness.

In a 2CO_2_ atmosphere, there are several major regions where the sea-ice thickness is reduced by more than 2 m (figure 4*c*), and again to a lesser extent in the MCB case (figure 4*d*). It is therefore likely that the loss of ice may occur at a greater rate than current model predictions—30 per cent (as cited earlier) for the double carbon dioxide scenario, consistent with Winston [22]. With MCB seeding switched on, there remains an increase in sea-ice thickness at the North Pole, but a marginal change at the South Pole.

In summary, taking both the ice fraction and depth characteristics together, seeding significantly reduces the sea-ice fraction loss during the summer months. The southern minima reduction in sea-ice fraction is smaller than in the Northern Hemisphere. The increase in sea-ice thickness near the pole in the geoengineered scenario does not alter the albedo of that region. In the Northern Hemisphere MCB run, there is an increase in sea-ice fraction to the north of Siberia, which increases the albedo relative to the control. The changes in ice cover fraction are consistent with those of Rasch *et al.* [6], but the reduction of the Southern Hemisphere ice fraction is significantly smaller in our calculations. The simulations indicate that our seeding with *n*=375 cm^−3^ increases ice extent in the double carbon dioxide scenario. Results from seeding all the suitable oceanic areas, not presented here, produce a further enhancement of planetary albedo and growth of polar ice cover compared with the control scenario.

## 3. High-resolution cloud modelling

### (a) Why is high-resolution cloud modelling essential for marine cloud brightening?

Despite considerable improvements over the last decade (especially in forecast models, e.g. Abel *et al.* [23]), marine boundary layer (MBL) clouds remain poorly represented in global models [24] and as such are a critical bottleneck in improved estimation of climate sensitivity in global models [25]. The difficulty in representing MBL clouds in global models is that many of the processes that control these clouds (e.g. turbulence, entrainment, heat and moisture transports, and precipitation) are not explicitly resolved owing to poor model resolutions, and, instead, need to be parametrized.

Additional aerosols injected into the MBL modify clouds through aerosol indirect effects that lie at the heart of the cloud-brightening scheme. The first indirect effect—the increase in cloud top reflectivity to incoming solar radiation—was proposed by Twomey [26]. It describes how the cloud albedo increases owing to an increase in aerosol number in the absence of any macroscale changes in clouds (i.e. changes in cloud cover, thickness, liquid water content, etc.). However, it is now known that a number of changes in the macrophysical properties can occur as a result of changes in cloud microphysical properties. Reduction in droplet size as a result of increasing droplet number may suppress precipitation [13], which may lead to a further enhancement of cloud albedo by increasing boundary layer moisture or a reduction of cloud albedo through increasing entrainment of dry free-tropospheric air [27–29]. Recent *in situ* and satellite remote-sensing observations indicated that precipitation in MBL clouds seems to be the rule rather than the exception [30–32]. Changes in precipitation induced by aerosols can drive mesoscale circulations that determine cloud structures [33–35]. When considering the deployment of cloud brightening over large tracts of the world’s oceans, it will therefore be essential to better understand how precipitating clouds respond to increases in CCN.

Other secondary effects may occur as a result of cloud microphysical changes such as changes to the evaporation and condensation rates in cloud [36] and changes in entrainment driven by reduced sedimentation rates of cloud droplets near cloud top [37]. The ultimate cloud albedo response is a result of an interaction among numerous complex processes (see the review by Stevens & Feingold [15]). All these associated effects and processes make the parametrization of MBL clouds in global models a real challenge.

High-resolution cloud modelling, including large-eddy simulation (LES; with tens of metres horizontal grid spacing) and cloud-resolving modelling (CRM; with hundreds of metres of horizontal grid spacing), can explicitly resolve processes that control clouds and aerosol–cloud interactions at different levels of detail, which are essential for the idea of cloud brightening. It provides a useful tool that can help improve process-level understanding and evaluation of the MCB scheme. It can also provide a necessary and critical test of the efficiency of cloud-brightening strategies.

### (b) The current state of high-resolution cloud modelling for marine cloud brightening

Ship tracks (i.e. bright cloud lines formed around ship-emitted aerosol particles in the MBL as seen in visible satellite imagery) have served as striking examples of aerosol effects on brightening MBL clouds and as inadvertent experiments for understanding aerosol–cloud processes relevant to MCB. They are brighter than adjacent clouds owing to more numerous but, on average, smaller cloud droplets and possibly more cloud water. Inspired by the formation and evolution of ship tracks, Rosenfeld *et al.* [38] proposed to enhance MBL cloud albedo by switching open-cell marine stratocumulus clouds (i.e. dark cellular regions ringed by bright cloud edges) to closed cells (i.e. bright cloud cells ringed by darker edges). The two distinct cloud cellular patterns occur very often over oceans but have very different overall albedo. It has been shown that aerosols can modify the formation/transition of the two cloud patterns. Therefore, putting the study of ship tracks in the context of open- and closed-cell MBL clouds is of particular interest from the perspective of MCB.

LES and CRM have long been devoted to studying MBL clouds and aerosol–cloud interactions. To date, however, only a very few LES and/or CRM studies have explicitly attempted to simulate the effects of seeding low marine clouds from a moving point source (e.g. ship emission), as proposed by the MCB scheme. Using high-resolution cloud simulations, Wang & Feingold [33] demonstrated that the concentration of CCN in the boundary layer can help determine whether marine stratocumulus clouds adopt open- or closed-cellular structures, with significant implications for overall albedo. More relevant to cloud brightening, however, is that, once the cloud cellular structures are established, they tend to resist change and do not necessarily follow conventional aerosol indirect effect responses [34,35]. The numerical model they used is the weather research and forecasting (WRF) model with a new treatment of aerosol–cloud interactions. Simulations were performed over rather large domains (60×60 km^2^ and 60×180 km^2^) with a grid spacing of 300 m (horizontal) and 30 m (vertical). The simulations fall into a realm between traditional LES and CRM. Nevertheless, useful and realistic results on cellular cloud formation and resolved aerosol–cloud processes have been produced [33,34].

**Figure 5.**
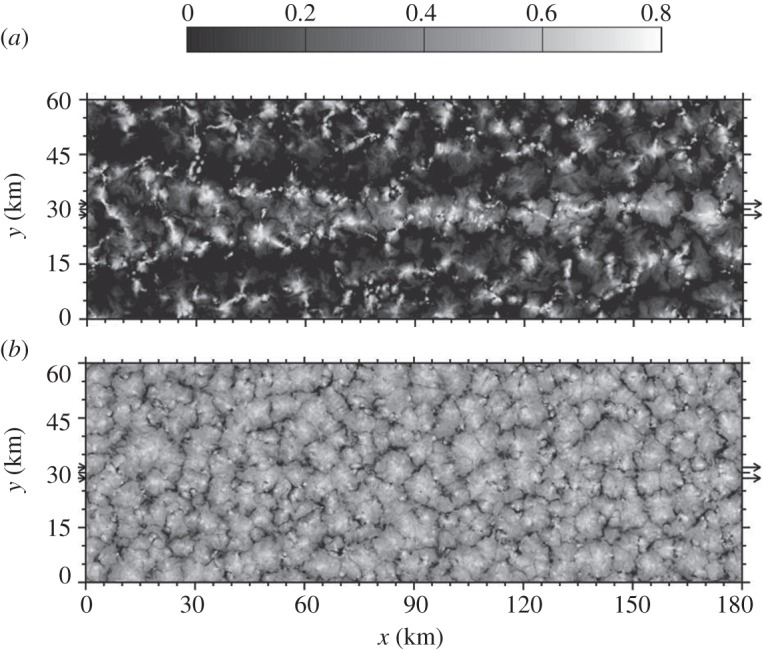
Snapshots of the cloud albedo field when ships pass through the domain once from *x*=0 to 180 km, about 7 hours after the start of the simulations. The background aerosol number concentration varies linearly from a lower bound at *x*=0 to an upper bound at *x*=180 km; (*a*) clean case 60–150 mg^−1^ and (*b*) the polluted case 210–300 mg^−1^. Arrows indicate the direction of movement of the ships and the band of ship plumes emitted near the surface. Details on the model and experimental set-up can be found in Wang & Feingold [33,34].

Meteorological conditions and cloud properties measured over the northeast Pacific off the coast of California were used to initialize and constrain the model simulations. In addition, the initial CCN number concentrations can be varied to modify rain production in the modelled clouds, through which the aerosol can determine cloud cellular structures. Additional ship-emitted aerosols can further modify existing clouds. For example, figure 5 shows the impact of ship emissions on clouds in both clean/precipitating and polluted/non-precipitating environments. An open-cell structure forms in the precipitating case. A ship track is clearly visible in the cloud albedo field (figure 5*a*) for the clean/precipitating case as would be expected even with Twomey’s argument. However, there are subtle changes in the cellular structure along the track from the plume head to tail, indicating that the interactions among ship-emitted CCN, clouds and precipitation vary with time. As revealed by Wang & Feingold [34], precipitation is suppressed most in the central section of the track, whereas new and sometimes stronger precipitation develops some distance behind the plume head, resulting in restoration of the open-cell structure. This, together with the less reflective dark regions close to the lateral boundaries of the ship track, is caused by a mesoscale circulation owing to dynamical feedbacks associated with the initial suppression of precipitation along the ship track. Convergent branches of the local circulation, located in the lower boundary layer over the track, pump moisture from the regions adjacent to the track and divergence in clouds helps dilute the ship-emitted CCN. Quantitatively, cloud albedo along the ship track was enhanced by 0.08 (averaged over 10 hours; [34]), while the domain average albedo was only 0.015 higher than that of un-seeded clouds. The dark edges (figure 5*a*) partly cancelled out albedo enhancement along the ship track.

Although ship emissions are the same in the polluted/non-precipitating case, the ship track in figure 5*b* is nearly invisible because the relatively small enhancement in cloud albedo (an average of 0.02; 4.3% relative to the domain average) is masked by the highly reflective cloud background. In addition, there is no dynamical feedback associated with the interaction between the CCN perturbation and precipitation because the polluted cloud is non-precipitating. When averaged over the entire domain, the albedo enhancement in the polluted case becomes even smaller, 0.005.

Formed in a sufficiently polluted environment, closed cells as shown in figure 5*b* are over two times brighter than open cells in figure 5*a*. The most ideal outcome of cloud seeding/brightening would be turning open cells into closed ones, as suggested by Rosenfeld *et al.* [38]. Can an influx of aerosols close open cells? There is no clear and firm answer yet. Numerical experiments conducted by Wang & Feingold [34] suggest that, once the open-cell structure is formed, simply adding more aerosol particles, even in large quantities, does not necessarily transform it to a closed-cellular structure.

These high-resolution modelling studies suggest that seeding marine stratocumulus clouds, especially those that are precipitating, is more complicated than that predicated by the conventional aerosol indirect effects. The albedo response depends on meteorological conditions, background aerosol concentrations and seeding strategy, which together determine the spatial distribution of injected aerosols and cloud properties, whether the clouds precipitate and therefore whether precipitation–suppression feedbacks can operate. Using the same numerical model (WRF) and similar model settings, Wang *et al.* [16] describe more details of different meteorological and microphysical scenarios in this context, providing implications for experimental strategies to adopt in the field.

### (c) Future of high-resolution cloud modelling in marine cloud brightening

The inability of global models to adequately represent MBL clouds and the unresolved complexities of aerosol–cloud–precipitation interactions in such clouds are major limitations in the assessment of the Earth system response to future changes in climate, regardless of whether the change was caused inadvertently or was deliberately engineered. Improving our knowledge of such processes should therefore be a major research goal, which relies much on high-resolution cloud modelling (e.g. LES and/or CRM). We suggest that any future research programme on cloud brightening should include a high-resolution cloud-modelling component. More work is necessary to understand how ship tracks such as those shown earlier form in response to idealized seeding strategies under different meteorological conditions and with different aerosol background states [16]. Beyond this, high-resolution modelling should be used to assess the interaction of plumes from multiple seeding platforms such as those that would be necessary to deploy cloud brightening as a geoengineering scheme, regionally or globally. We currently have little idea on how clouds would respond to multiple aerosol plumes beyond what Wang *et al.* [16] have shown, and yet figure 5*a* and Wang *et al.* [16] suggest that there are regions where the induced mesoscale flows in the boundary layer act constructively and other regions where they destroy clouds, producing unintended consequences that reduce expected albedo response. In their 1 day simulations, Wang *et al.* [16] found that the injection strategy is critical in determining the spatial distribution of the injected aerosols and there is a case-dependent effective timing of injection during the diurnal cycle of marine stratocumulus. Longer time and more comprehensive high-resolution cloud modelling can be used to examine how rapidly induced aerosol perturbations from seeding are removed by coalescence scavenging and dilution from entrainment of free-tropospheric air, providing guidance on the timing and duration of injection. These issues will be particularly pertinent when designing field experiments to test critical aspects of cloud brightening.

## 4. Detailed modelling of the effects of sodium chloride spray on cloud–albedo change

The purpose of this section is to explore the range of dry salt masses and concentrations that are most effective for altering the albedo of MBL clouds.

### (a) Explanation of model and set-up of run

We have used a new cloud parcel model with size-resolved or bin microphysics that has been developed at Manchester, UK, and is called the aerosol–cloud and precipitation interactions model (ACPIM) [39]. The work we have carried out here builds on that previously reported in Bower *et al.* [3]. In their work, the composition of the background aerosol size distributions and that of the added aerosol particles was prescribed to be sodium chloride. The added particles also had a single monomodal size. In this work, the size distributions of the background aerosol distributions are the same as in Bower *et al.* [3] but are composed of ammonium sulphate to which sodium chloride particles are added in a mode of finite width to replicate more realistically the size distributions of particles that can be generated by the spray-production techniques described in §5*b*. The lower limit of added salt particle mass in Bower *et al.* [3] was 10^−18^ kg, sufficient to cover the range of dry particle sizes under consideration by Salter *et al.* [4]. However, the range of the mass of added salt particles has now been extended to smaller sizes, to encompass the size range that can be produced using the Taylor cone technique (described later), which produces dry salt particles in the mass range of approximately from 3×10^−20^ to 5×10^−19^ kg. Note that in the atmosphere it is well known that the dry salt particles would take on water and swell to larger physical sizes as a result of the Raoult effect.

The parcel model version of the ACPIM used here activates aerosols in a sectional way. The ACPIM also uses a more thorough description of the thermodynamics of the aerosol [40] than was present in the NEATCHEM model used in the Bower *et al.* study. Three sets of model runs were performed with ACPIM; in each set of the runs, the control corresponded to running the model with a ‘background’ aerosol size distribution measured in three different air masses (the ‘clean’, ‘medium’ and ‘dirty’ distributions used in Bower *et al.* [3]). Clean corresponds to a total number concentration of approximately 10 cm^−3^; medium approximately 260 cm^−3^; and dirty approximately 1000 cm^−3^.

Koehler theory was used to determine the equilibrium vapour pressure of the aerosols [40] in the background size distribution of particles (composed of (NH_4_)_2_SO_4_). The initial relative humidity, pressure and temperature in the model were set to 95 per cent, 950 hPa and 283.15 K, respectively, and the model was run until the parcel was lifted to a total height of 250 m. These conditions are typical of stratocumulus clouds observed in the southeast Pacific Ocean that have large spatial coverage (figure 1). Typically, this generated a cloud base (i.e. saturation level) approximately 75 m above the starting level and hence a cloud approximately 175 m deep, allowing comparison with the results of Bower *et al.* [3]. Future work will look at the sensitivity of the addition of aerosols to deeper (i.e. more optically thick) clouds, although (as in the studies of Bower *et al.* [3]) the trends in albedo differences produced are expected to be similar. These simulations were repeated for different prescribed vertical wind speeds of 0.2, 0.5 and 1.0 m s^−1^ to represent the typical range of updraught speeds found in marine stratocumulus. Sensitivity tests were then performed investigating the effect of adding a lognormal mode of aerosol to the background ammonium sulphate aerosol distributions to simulate the spread in sizes expected from the droplet spray technique. The composition of the particles in the added aerosol mode was NaCl, and their equilibrium vapour pressure was obtained from Koehler’s theory.

The parameters varied in these tests were the total number of added aerosol particles, *n*_add_, and their dry salt mass *m*_*s*_. The parameter values used were *n*_add_=0, 30, 300 and 1000 cm^−3^ and *m*_*s*_=1×10^−20^, 3×10^−20^, 7×10^−20^, 1×10^−19^, 3×10^−19^, 7×10^−19^, 1×10^−18^, 1×10^−17^, 1×10^−16^, 3×10^−16^, 1×10^−15^ kg (or 1.06×10^−2^, 1.53×10^−2^, 2.03×10^−2^, 2.29×10^−2^, 3.30×10^−2^, 4.37×10^−2^, 4.92×10^−2^, 1.06×10^−1^, 2.29×10^−1^, 3.30×10^−1^, 4.92×10^−1^ μm dry aerosol diameter, respectively). This range was chosen not necessarily because it spans the range capable of being produced by the current spray generators (see §6), but because we wanted to determine where the main sensitivities lie. Addressing this will inform future spray generator development. The added lognormal mode was specified to have a median diameter equal to that of the added dry salt particles, that is
4.1
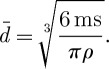

In all cases, the standard deviation of the mode was specified to be 0.25. The parameter values listed totalled 41 runs per prescribed updraught value, a grand total of 369 runs (including runs with *w*=1.0 m s^−1^, which lead to smaller particles becoming activated; however, the results are essentially similar to the lower updraught cases, so they are not presented here). In principle, each of the spray techniques will probably yield its own unique size distribution of NaCl particles, but it is not clear yet what these are. Preliminary results show some sensitivity to the mode width; so it is intended to further investigate this in order to inform spray technology engineers as to what tolerance is acceptable vis-à-vis this parameter.

In order to calculate the albedo for the simulation, we first calculated the volume extinction coefficient, *β*(*z*), by integrating the product of the total cross-sectional area of the particles by their scattering efficiency (approximated as 2 in this size regime, which is a reasonable approximation—see fig. 9.21 of Jacobson [41]),
4.2
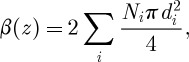

where *N*_*i*_ and *d*_*i*_ are the number concentration and diameter of the particles in bin *i*, and the sum is over every model size bin and each height level in the model. The solar optical depth, *r*, is then calculated by integrating the volume extinction in the vertical,
4.3
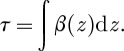

The approximate broadband albedo, *A*, is then calculated using the formula (see equation 24.38 of Seinfeld & Pandis [42])
4.4
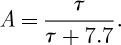

We report the total albedo change in this study that contains contributions from the direct effect and indirect effect. The direct effect is small when compared with the indirect effect in these calculations, and its magnitude will depend on the amount of aerosol and the humidity.

### (b) Results from model runs

**Figure 6.**
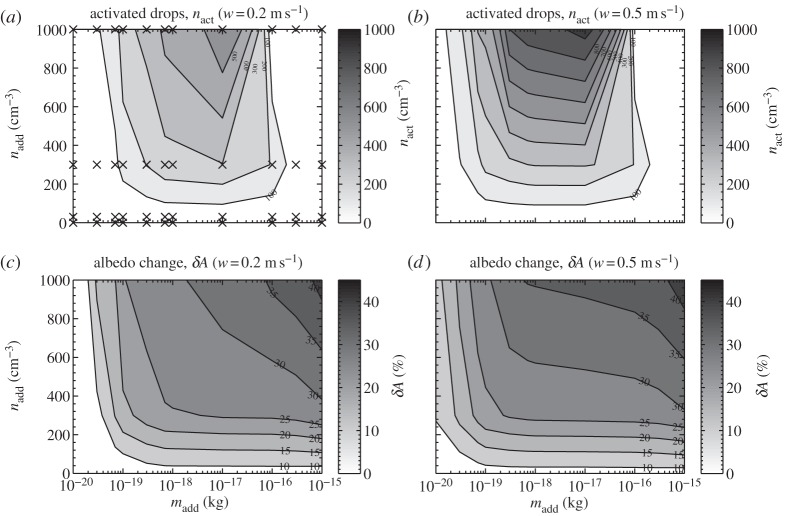
Summary plots for the clean air mass. The number of activated drops without the addition of NaCl were 8.8 and 9.8 cm^−3^ for *w*=0.2 and 0.5 m s^−1^, respectively. (*a*) A contour of the number of activated cloud drops when a distribution of NaCl aerosols of different total number and median mass are added to a rising parcel moving at 0.2 m s^−1^. The masses added are on the *x*-axis, whereas the corresponding number added is on the *y*-axis. Plus signs denote the different runs used to calculate the contour plot; (*b*) same as (*a*) but for an updraught of 0.5 m s^−1^; (*c*) the difference in the albedo between the control run and the run with the indicated aerosol added (*n*_*add*_, *m*_*add*_), in units of per cent, of the clouds resulting from seeding; (*d*) same as (*c*) but for 0.5 m s^−1^. Please refer to initial conditions in text for dry diameters corresponding to added dry particle masses.

Figure 6 shows results in the case where the background ammonium sulphate size distribution is taken from that measured in a ‘clean air mass’ [3]. This case represents the most pristine conditions we might expect to find in the maritime boundary layer. Concentrations in the medium case are slightly higher than found over the southeast Pacific (e.g. during the recent VOCALS experiment). The dirty case is very polluted. For the clean case, it can be seen (figure 6) that adding NaCl particles of dry mass less than approximately 1×10^−19^ kg results in no change to the cloud drop number because these particles have too high curvature and too low solute mass to be active CCN. Adding particles of dry mass greater than approximately 1×10^−16^ kg results in aerosols not activating to form cloud drops (figure 6*a*,*b*). However, the added sodium chloride aerosols, while not ‘classically’ activating (to form cloud drops), still take on appreciable liquid water, swelling to sizes approaching approximately 10 μm. The result of this is a thick haze having high extinction of solar radiation and hence a high albedo, as can be seen from figure 6*c*,*d*. The pre-existing ammonium sulphate aerosols have their activation suppressed. Between 1×10^−19^ and 1×10^−16^ kg dry mass, we are able to alter the modelled cloud drop concentration very effectively by changing the number concentration of added aerosols. Although the addition of NaCl particles of mass greater than 1×10^−16^ kg results in no aerosols being activated as CCN, the swelling of these aerosols still has the desired effect of increasing ‘cloud’ albedo, regardless of whether they are activated. However, adding aerosols of this size or greater (which are effectively giant CCN) may result in undesirable effects such as the more efficient production of rain; an effect that will be investigated in future work). The maximum change in albedo for the clean air mass is around 0.4, rising from an albedo of 20 per cent for the control to 60 per cent for the case in which high concentrations of large NaCl particles have been added.

The pattern of aerosols not strictly being activated but still contributing to albedo difference was observed in both the medium and dirty cases, so the plots of cloud drop number are not shown here.

**Figure 7.**
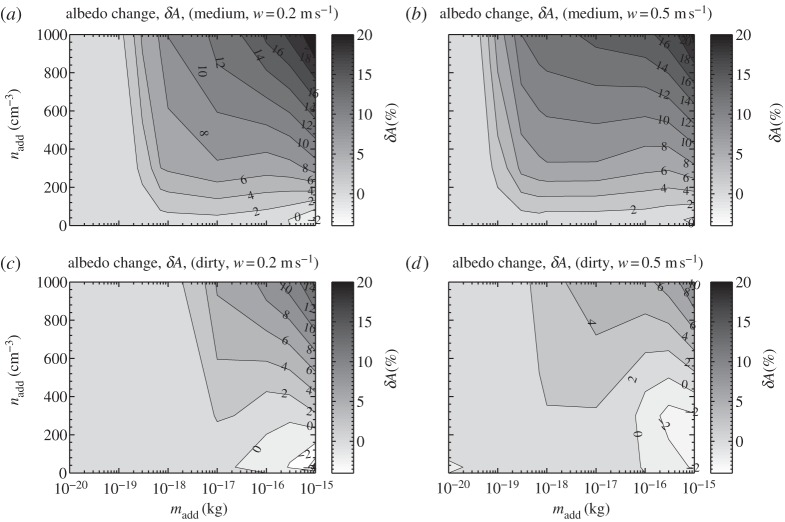
Summary plots of the albedo change for the medium and dirty air-mass cases. For the medium case, the number of activated drops without the addition of NaCl were 142 and 179 cm^−3^ for *w*=0.2 and 0.5 m s^−1^, respectively, whereas for the dirty case these were 358 and 639 cm^−3^ for *w*=0.2 and 0.5 m s^−1^, respectively. (*a*) The difference in the albedo between the control and the run with the indicated aerosol (*n*_add_,*m*_add_) for the medium case with 0.2 m s^−1^ updraught; (*b*) the same but for 0.5 m s^−1^; (*c*,*d*) the corresponding contours of albedo change for the dirty case.

Figure 7*a*,*b* shows the model results for the medium loading ammonium sulphate background air-mass case [3]. Qualitatively, the results are similar to the cleaner air-mass results except for two key differences: (i) the magnitude of albedo difference is about a factor of 3 smaller than in the clean case and (ii) for the lower updraught case (*w*=0.2 m s^−1^) adding relatively few large NaCl particles may actually reduce the albedo of the clouds by a small amount. The reason for this is that a few large NaCl particles are able to reduce the peak supersaturation in the rising parcel enough to reduce the number of cloud drops in the background spectrum that would otherwise activate to form cloud drops, but not enough to suppress activation entirely. This reduction in turn reduces the extinction of the clouds as there are fewer, larger particles than in the control case. Suppressing activation entirely (i.e. when adding many large NaCl particles) results in many large swollen aerosol particles and hence larger extinction, as can be seen from figure 7*a*,*b*. In the absence of seeding, the concentrations of cloud droplets generated in the background in the clean, medium and dirty cases were 8.8, 142, 358 cm^−3^, respectively, for an updraught of 0.2 m s^−1^, and 9.8, 180 and 639 cm^−3^, respectively, for an updraught of 0.5 m s^−1^.

Figure 7*c*,*d* shows the model results for the case in which there is a high concentration of background ammonium sulphate aerosol present, corresponding to a ‘dirty air mass’. Qualitatively, the results are much the same as for both the clean and the medium air mass cases. One difference is that the albedo of the clouds is now less susceptible to the inclusion of additional sea salt aerosol. There was little increase in cloud droplet number even when adding particles approaching 1×10^−18^ kg in mass, especially for the low-updraught case (not shown here).

In the medium and clean cases, a larger increase in cloud droplet number was found for the addition of NaCl aerosol of this or even smaller mass. The reason for this decreased sensitivity is that, in the dirty case, there are already copious (NH_4_)_2_SO_4_ particles present in the background aerosol to deplete the supersaturation at cloud base such that the NaCl particles of approximately 1×10^−18^ kg cannot be activated. Similarly, the point at which drops cease to be activated has also changed. In the previous cases, drops ceased to activate when NaCl particles of mass approximately 1×10^−16^ kg (or larger) were added. In this case, activation of additional drops ceases at a lower threshold sea salt particle mass (typically 7×10^−17^ kg or less). This is because the higher concentration of background aerosol contributes significantly to the reduction in supersaturation in the rising parcel of air, suppressing further activation. Another notable difference is that the maximum change in albedo that is achieved is considerably less than for the clean case, and slightly less than in the medium case too. More noticeable in this case is a region where a reduction in albedo occurs when adding relatively few large-mass NaCl particles.

### (c) Conclusions

The modelling suggests the following:

— The enhancement to the albedo is greatest for clean background conditions. This is consistent with previous work by Bower *et al.* [3].
— In the clean conditions, the albedo of the control case cloud was approximately 20 per cent, whereas for the case where many large NaCl particles were added it was approximately 60 per cent. This (factor of 3) difference should be easily observable in a field campaign. In the medium and dirty cases, these increases in albedo were a factor of 1.6 and 1.3, respectively. This corresponds to albedos in the control runs for the medium and dirty cases of 35 per cent and 45 per cent with the maximum absolute increases in albedo of 20 per cent and 12 per cent, respectively. The magnitude of these changes will vary slightly with cloud depth (although the trends will be similar), and this will be investigated in future work.
— The values of the albedo in the control runs are typical of observed stratocumulus clouds and are in the same range as those in the cloud modelling section (figure 5).
— For both the medium and dirty cases, a reduction in cloud albedo was found when adding relatively low concentrations of particles that have NaCl masses of approximately 1×10^−16^ and greater. This underscores the findings by Bower *et al.* [3] that, for efficient albedo enhancement, the added particles should have masses higher than almost all natural particles and be added in significantly greater numbers; however, current technology is unable at present to generate such large particles in significant concentrations (see §6). Nevertheless, this study has shown that adding smaller particles of 3×10^−19^ kg (0.033 μm) results in smaller, but still significant, albedo enhancement. Furthermore, adding particles of salt mass less than 1×10^−19^ kg in the clean and medium cases and less than 1×10^−18^ in the dirty case produced little change to the drop number.
— While the most efficient albedo enhancement is achieved by adding large NaCl particles, it should be noted that such large particles may also initiate rain that is detrimental to cloud brightening as it tends to reduce cloud lifetime [13]. This effect needs further investigation both with high-resolution models (see §3) and further parcel modelling.

When performing this study, we chose conditions to be relevant to those that seed aerosols would experience as they rise through a stratocumulus cloud layer in the southeast Pacific Ocean and hence we are limited as to the generality of our conclusions. We expect that, in general, the results would not be too different in all marine stratocumulus clouds. However, it is noted that the scheme will not be as effective in marine stratocumulus clouds that are close to significant sources of anthropogenic aerosol.

## 5. Engineering steps to implement marine cloud brightening

### (a) Introduction

Previous sections have considered the science of cloud brightening by increasing the CCN of marine stratus clouds (by way of a very fine, evaporating spray of sea water microdroplets) and the foreseen impact on global climate. In this section, attention is turned to what are seen as the two major technological challenges that have a vital bearing on the effectiveness: the time scale for development and the overall costs of the scheme. These are, first, how one might generate the mist of microdroplets of the desired size and spray-rate needed and, second, what strategy should be adopted for delivering the spray; for example, whether from mobile or stationary sources and, if from a mobile source (i.e. a ship), the type of vessel and the optimum means of propulsion.

In fact, both these aspects were considered, if not entirely resolved, by Salter *et al.* [4] and this section mainly concentrates on new developments. That paper had already concluded that sea-level dispersal of an evaporating spray had decisive advantages over the more direct approach of cloud seeding from aircraft and that, among the several alternative strategies, dedicated sea-going vessels propelled by Flettner rotors (which facilitated an unmanned operation) were the preferred technical choice as well as being, by a considerable margin, the cheapest and ‘greenest’ route. The performance of Flettner rotors had not, however, been examined for more than 80 years and thus in §5*c* our first results based on CFD of the dynamic performance of a single rotor are presented. This is preceded, in §5*b*, by an even more pressing issue—the approach to be used for producing the salt-water spray.

### (b) Electrohydrodynamic spray fabrication

We have explored experimentally a number of ways to produce sea water droplets that would be suitable for use in cloud brightening. The critical requirement is that their salt mass *m*_*s*_ be high enough that they can convert into cloud droplets at the supersaturation, *S*, occurring in marine stratocumulus clouds. *S* depends on updraught speed, and the properties of the air mass. Cloud modelling (described later) provides values of critical mass for a variety of relevant scenarios. They show that significant droplet formation and associated cloud albedo increase can occur for *m*_*s*_ values down to about 5×10^−20^ kg. Hence, the initially sprayed droplets, drying to a quarter of their initial size, should minimally be of the order of 150–200 nm diameter. For energy efficiency, it is advantageous to make the droplets close to this lower acceptable limit, for it is the number of suitable nuclei formed, not the amount of water sprayed, that is important. The smaller the size of the droplets capable of inducing activation, the smaller the required amount of spray with its associated energy and evaporative air cooling.

We investigated the performance of standard commercial nozzles that are used in fogging systems, toroidal vortex-based nozzles, colliding water jets, ultra-high-pressure nozzles (345 MPa) and Rayleigh-mode jet break-up from micromachined and radiation-track apertures. These experiments will be detailed in another publication, but, so far, none has produced encouraging results.

The best results to date were obtained using Taylor cone-jets [43], drawn from porous tips. Upon application of a voltage to a capillary containing a fluid, the most interesting of the spraying modes is the cone-jet, i.e. a cone terminating in an emerging jet. The 49.3^°^ half-angle cone first described by Taylor is well understood, but the jet description is much more complex, particularly for high-conductivity liquids such as sea water. Analysis by De la Mora [44] and Gañán-Calvo & Montanero [45] show that a critical radius (*r*_*i*_) exists, defined by the flow and the dielectric relaxation constant of the fluid. A highly charged jet of approximate radius 0.2 *r*_*i*_ emerges and breaks up monotonically, similar to that of an uncharged Rayleigh jet. Each drop is often accompanied by a satellite drop having a mass a few per cent of that of the parent drop.

**Figure 8.**
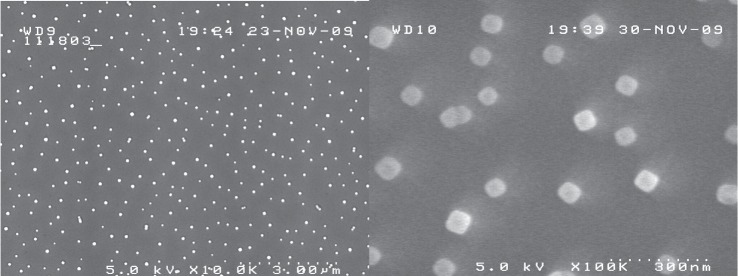
SEM images of salt particles from salt-water cone-jets at different magnifications.

Figure 8 shows scanning electron microscope (SEM) images, at different magnifications, of salt particles produced by a Taylor cone emanating from a porous tip, collected on a silicon wafer at a 5.4 kV potential, a current of 0.2 μA with a flow of 5.6 nl s^−1^, using 540 ppm of surfactant. The surfactant lowers the instability threshold below air breakdown, eliminating the corona discharge that destroys the uniformity of the particle distribution. The average size of these crystals is of the order of 75–85 nm, having a mass of approximately 10^−18^ kg, suitable for the intended purpose. The droplets evaporate before they reach the silicon wafer 2 cm away. This near instantaneous evaporation of the droplets is due to their emergence from the jet with velocities approaching the speed of sound, and the heating that takes place in the cone itself [46]. These crystals readily convert at a supersaturation of 0.5 per cent, achieved by cooling the wafer with a thermoelectric chuck in an enclosed environment.

Although each cone-jet produces a very large number of droplets (of the order of 10^8^–10^9^ s^−1^), scale-up requires 10^8^ jets to reach roughly 10^17^ nuclei s^−1^ per sprayer. Small arrays of porous tips work well, but the overall size would be prohibitive. Various efforts have been made to mass produce cone-jet capillaries and associated extraction plates. Perhaps the most relevant for our purposes is the work of Deng *et al.* [47], who described the micromachining of silicon capillaries and extraction plates, alignment methods and the production of arrays with up to 331 nozzles, producing remarkably uniform spray, with only a few per cent of size deviation. The density of the capillaries exceeds 100 mm^−2^, suggesting approximately 1 m^2^ in total for the nozzle array that is technically feasible by tiling.

As a low-cost alternative, we have pursued the use of holes in low-dielectric polymeric materials (PEEK, polyimide, PMP) in place of capillaries. This approach was first outlined by earlier studies [48,49]. This technique lowers power consumption, and the fabrication of holes is significantly easier than that of capillaries. These holes must have a large aspect ratio in order to avoid interaction between adjacent holes. The problem can be overcome by using a dielectric thin film (50–75 μm) attached to a porous block that provides flow impedance, isolation and filtration at the same time. Such arrays may then be made by fast and inexpensive laser drilling systems. To fabricate prototypes we were able to make use of a Samurai UV marking system (courtesy of DPSS Lasers Inc., Santa Clara, CA), capable of drilling 50 000 holes s^−1^. Hence, the drilling of 100 million holes is a manageable task, requiring a hole every 100 μm over a total area of 1 m^2^.

The other requirement [48] is that the water needs to be confined at the rim of each individual hole, or jets will coalesce. To this end, it has been found that the dielectric material needs to be made superhydrophobic, i.e. the fluid contact angle must be in excess of 150^°^ [11]. Polyimide films were made superhydrophobic by plasma etching with oxygen, yielding a rough surface, followed by plasma deposition of a 20 nm fluorocarbon film. The combination gives rise to the desired surface properties, with water contact angles approaching 160^°^. However, the surfactant needed to obtain reliable cone-jet spraying of sea water lowers the contact angle to values that are unacceptable. Using films made at the Stanford Nanofabrication Facility or supplied by commercial sources (Repellix; Integrated Surface Technologies, Inc., Menlo Park, CA), we were unable to find a combination of surfactant and robust surface preparation that satisfies all the requirements.

The surfactant requirement can be eliminated by a number of methods: increased ambient pressure or smaller apertures. If the ambient pressure is raised slightly (20%), the air breakdown field increases. With slight over-pressurization, corona discharge disappears and there is no need for surfactants. Raising the pressure causes airflow through the extraction apertures and, while the flow through each hole is small, an array of 100 million holes demands a substantial amount of power. The flow of air is of course beneficial in helping the passage of the droplets through the extractor holes. Likewise, when the capillary holes are made smaller than 10 μm, the air breakdown field (increasing with decreasing jet radius) is at all times higher than the field over the cone itself; so no air breakdown occurs here either.

In summary, the fabrication of large arrays of Taylor cones, either by silicon micromachining or by laser drilling in dielectric sheets, seems quite feasible although no such large arrays have yet been constructed. It is estimated that for an array of 100 million holes, roughly 1 m^2^ in size, the electrical power requirement would be less than 100 kW. If airflow is used, then there would be an additional requirement of 270 kW for pneumatic power. Because more than 90 per cent of the electrical power ends up as droplet kinetic energy, it can probably be partially recovered by reverse induction using a Kelvin generator arrangement.

As a simpler alternative, we are exploring the spraying of sea water at or near its critical point. In this regime, water has little or no surface tension and a gas-like viscosity and hence should produce fine dispersions. This has been demonstrated in the pharmaceutical industry with the spraying of supercritical carbon dioxide containing dissolved therapeutic compounds. While the distributions resulting from this technique are bound to be wider than those from cone-jets, the resultant particle distribution can on occasion be quite uniform [50]. Results of this investigation will be reported later.

We used the model described in §4 to examine in more detail the conditions under which the electrohydrodynamic spraying technique could produce albedo change values of significance (i.e. not less than about 0.06, or 6%). We present in table 1 the change in albedo (for each air mass) that could be achieved by adding 1000 cm^−3^ of NaCl particles of mass within the range currently achievable by this technique (i.e. up to about 10^−19^ kg). We present only the 1000 cm^−3^ results because our model results showed that this led to the maximum change in albedo. It can be seen that the technique can result in large albedo change in clean air masses. For the medium-polluted air mass, only particles of salt mass larger than or equal to approximately 3×10^−19^ kg result in an albedo change that may be significant for offsetting warming by carbon dioxide, whereas for the dirty air mass all salt masses result in negligible albedo change.

**Table 1.** Δ*A* values (in %) achieved in the 0.2 m s^−1^ updraught case for runs where 1000 particles of NaCl per cm^3^ were added in the range 1×10^−20^ to 3×10^−19^ kg.

air mass	mass (kg) 1×10^−20^	3×10^−20^	7×10^−20^	1×10^−19^	3×10^−19^
clean	3.9	16	20	25	28
medium	9.1×10^−3^	3.8×10^−2^	5×10^−1^	1.4	6.5
dirty	1×10^−2^	2.3×10^−2^	4.7×10^−2^	6.5×10^−2^	1.8×10^−1^

This highlights that, in very clean clouds, the electrohydrodynamic spray technique is feasible. However, in the medium and in the dirty air masses, in particular, we would probably need to produce larger particles of (around 1×10^−18^ kg), as suggested by figure 6.

### (c) Computational fluid dynamics studies for optimizing the Flettner propulsive system

#### (i) Scene setting

The most direct and probably the most obvious route for supplying the additional CCN would seem to be by directly seeding the microparticles from aircraft flying below the bases of the marine clouds to be brightened. Our earlier work on MCB [4,5] had firmly concluded, however, that sea-level injection of microdroplets of sea water would be as effective while offering major environmental and cost-saving benefits. Among the sea-level options for seeding the marine clouds, fixed spraying locations from anchored platforms would have to be too numerous to provide a reasonable coverage of the most suitable regions and their servicing at sea would be both hazardous and expensive. While the spraying equipment *could* be installed on regular cargo vessels as they plied the oceans, Salter *et al.* [4] concluded that it was better to have a vessel—or, rather, fleet of vessels—dedicated to the task of cloud seeding. Given the unusual role that these craft had to play, however, it was imperative that the ship’s design be open to possibly radical innovations. The most important of these was the proposal that the vessel should be propelled not by conventional diesel engine-powered propellers nor by sails, but by Flettner rotors.

A Flettner rotor (named after its inventor, Anton Flettner) is a vertically mounted cylinder that may be rotated about its axis by an external power supply. When air flows past it, the cylinder rotation creates a force (the Magnus force) at right angles to the air flow that propels the vessel on which the cylinder is mounted. The rotor thus plays the same role as the sails on a yacht but the thrust levels attainable are far greater than for a sail of the same area; moreover, the control of such vessels is very much simpler (without the complex rigging of a sail and with far superior manoeuvrability). This latter feature makes vessels powered by Flettner rotors ideal for unmanned, radio-controlled operation, a measure that clearly brings enormous savings in costs for it dispenses with the need for a crew and the associated multi-faceted support infrastructure. Moreover, it has been estimated [4] that the cost of providing the power to spin the rotor is an order of magnitude less than that required for a screw-driven vessel of comparable size sailing at the same speed. The fact that *that* speed would usually be less than half that of a diesel-powered craft in normal operation is immaterial for the purpose of cloud seeding.

**Figure 9.**
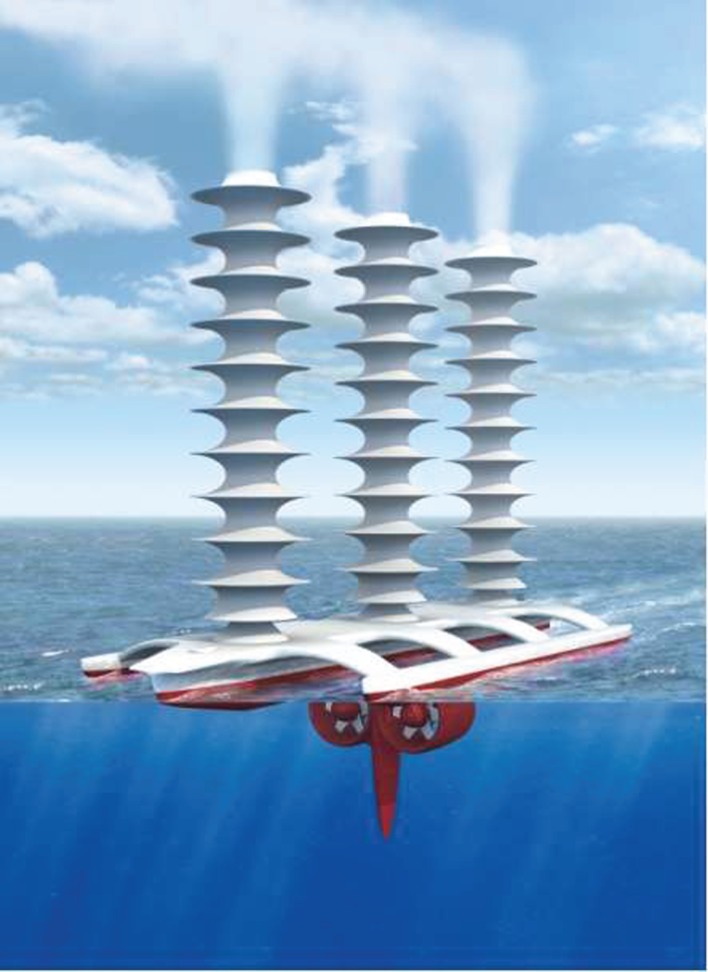
Artist’s impression of a Flettner rotor ship. Reproduced from Gadian *et al*. [51]. (Online version in colour.)

An artist’s impression of such a vessel is shown in figure 9. While the original Flettner vessel that crossed the Atlantic in 1926 was propelled by two purely cylindrical rotors, in the conceptual design of the cloud-seeding craft shown in the figure, the rotors have a number of discs mounted along their length. In fact, Thom [52] carried out a number of wind-tunnel experiments that suggested that the inclusion of such discs markedly improved rotor performance at high spin rates. Inevitably, however, the scope of that experimental exploration was limited and was certainly not conceived as contributing to the particular requirements of the cloud-seeding craft. Moreover, nearly 80 years on, as in so many areas, computer simulation (while not replacing the need for experiments) has made it feasible to explore a wide range of flow conditions and rotor geometries relatively rapidly and to provide far greater detail than any experiment. Here, therefore, our first results of applying CFD to the Flettner rotor problem are presented.

The first major computational study into the behaviour of flow past a rotating (bare) cylinder was undertaken by Mittal & Kumar [53] (hereafter M&K). While that study was limited to laminar flows at a Reynolds number, 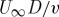 (5.1),^1^ of 200 (i.e. two or three orders of magnitude below those that would be encountered in an actual cloud-seeding vessel), their results revealed a potentially worrying feature with a major bearing on the present research. Over a limited range of rotation rates (relative to the wind speed), the flow around the cylinder experienced large-scale temporal periodicities that produced highly undesirable variations in drag and lateral forces on the cylinder. If these were present under operational conditions in the cloud-seeding vessel they would, *inter alia*, have a seriously adverse effect on the lifetime of the rotor and its support mechanisms. Thus, in the exploratory studies presented later of turbulent flow past the rotor at Reynolds numbers typical of operating conditions, such flow instabilities have been a major feature to watch out for.

#### (ii) Numerical and physical model

**Figure 10.**
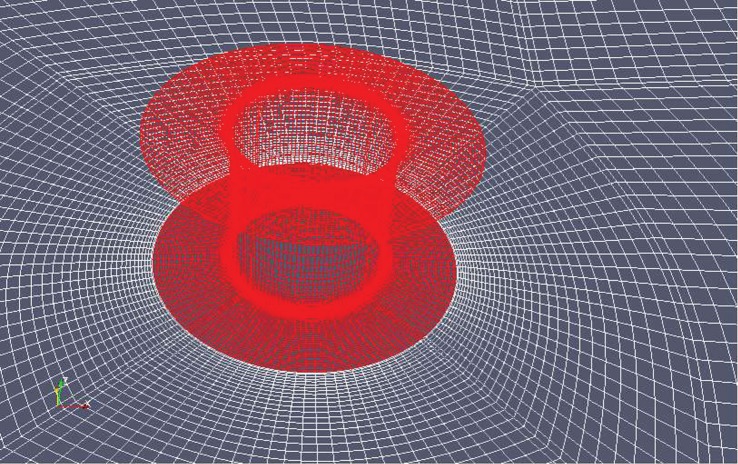
Details of the multi-block, non-orthogonal mesh. (Online version in colour.)

Computations have been performed using an in-house CFD solver, STREAM [54], to examine the flow around rotating cylinders with and without Thom discs. For these tests, the discs have been taken as flat annular plates of diameter twice that of the cylinder, axially spaced, one cylinder diameter apart. (This corresponded with the spacing shown in figure 9, although in Thom’s original tests the disc diameter was three times that of the rotor and the axial spacing just half the rotor diameter.) The results presented here have been obtained using a multi-block, non-orthogonal grid (figure 10) of around 0.75 M cells, covering a domain extending far enough from the cylinder for boundary effects to be negligible, and extending vertically from one disc to the next, as shown in figure 10. A uniform ‘wind’ velocity was specified around the inlet part of the outer boundary and zero-gradient conditions on the outlet. Symmetry conditions were applied along the two boundaries normal to the cylinder, and no-slip conditions, via ‘wall functions’, were applied at the disc and cylinder surfaces. For comparison, simulations were also performed for a bare cylinder (i.e. without discs).

For most of the test cases, the effects of turbulence were represented by a conventional *k*–*ε* linear eddy viscosity model with standard log-law-based ‘wall functions’ to provide the wall boundary condition. Some runs have, however, been made using more advanced stress-transport turbulence models and wall-function treatments (summarized, for example, in Craft *et al.* [55]). Although there are some modest quantitative differences in results between the different modelling approaches, cross checks show very similar trends, and because of the two- or threefold time penalty with the more elaborate model, most results were obtained with the simpler eddy viscosity scheme.

#### (iii) Initial computational results

To validate the procedure, purely laminar flow around a bare rotor for a Reynolds number of 200 was examined, corresponding to the case studied by M&K. In agreement with their results, the present computations confirmed that the Karman vortex street, present behind non-rotating cylinders, disappeared for dimensionless rotation rates, 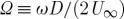, greater than 2 (where *ω* is the angular velocity of the cylinder). Moreover, for a narrow band of rotation rates around *Ω*=4.4, longer period, large-amplitude oscillations developed, although by *Ω*=5 these also disappeared, again broadly in agreement with the M&K results. As noted already, a major question in the present context is whether these instabilities also arise in turbulent flow at the much higher Reynolds numbers commonly encountered for a Flettner rotor.

**Figure 11.**
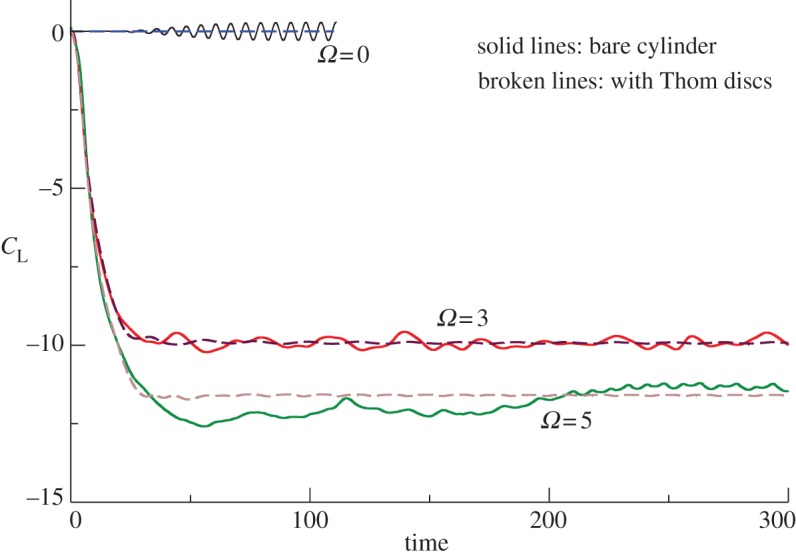
Predicted temporal evolution of experimental lift coefficient for turbulent flow at *Re*=8×10^5^ for a range of rotation. (Online version in colour.)

The presently predicted results for turbulent flow at *Re*=8×10^5^ are summarized in figure 11, which shows the dependency of the rotor’s lift coefficient, *C*_L_, on the non-dimensional rotation rate. For zero rotation, the bare cylinder results display the expected oscillatory pattern associated with the Karman vortex street. As the rotation is increased, these oscillations disappear, and the magnitude of *C*_L_ increases steadily. By a rotation rate of *Ω*=5, a lift coefficient of around 12 is predicted. Although rather less than half the corresponding value found for laminar flow, this is still sufficiently high to underline the value of the Flettner rotor as a propulsive device. A further point to note from the bare cylinder results is that the large-amplitude oscillations seen in the laminar flow calculations around *Ω*=4.4 were not detected in the turbulent case for the rotation rates examined. However, as can be seen from figure 11, at rotation rates above *Ω*=3, the solution did not exhibit an entirely steady behaviour, indicating that there are nevertheless unsteady three-dimensional structures present in the flow, although not in an organized, regularly repeating form.

Turning now to the effects of the Thom discs, the time histories of the lift coefficient, also included in figure 11, indicate that the mean values of *C*_L_ are not very different from those of the bare cylinder. A feature worth noting, however, is that, by including the discs, a much steadier flow field is achieved. For the case of no rotation, the Karman vortex street is suppressed, and a constant value of *C*_L_ (zero) is thus returned. At rotation rates of *Ω*=3 and 5, although there are some small undulations in the *C*_L_ time history, these are very minor (and fairly periodic) compared with the behaviour of the bare cylinder.

**Figure 12.**
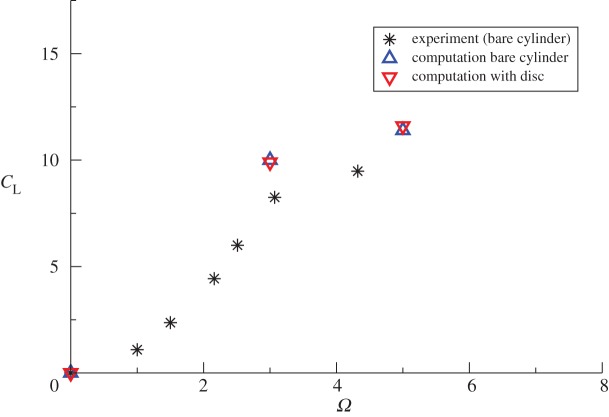
Mean lift coefficients for a bare cylinder and a cylinder with discs over a bare cylinder for a range of rotation rates. Measurements from Reid [56]. (Online version in colour.)

Figure 12 compares the predicted mean lift coefficient, as a function of rotation rate, with and without discs and the very early (but still among the most comprehensive) measurements of Reid [56] for a bare cylinder. The experimental data for the bare cylinder show a fairly rapid rise in *C*_L_ as *Ω* is increased from 0 to around 3, followed by a more moderate rate of increase thereafter. The present results broadly reproduce this pattern. The numerical results show values slightly higher than the measurements. As noted earlier, the calculations show only minor differences in average *C*_L_ values between the cases with and without Thom discs.

Finally, the question of whether large-amplitude periodicities may arise cannot yet be answered definitively. The computations of M&K and our own show that, in the laminar-flow regime, these instabilities appeared only over a very narrow range of spin rates. Preliminary turbulent flow studies have suggested that such oscillations may also occur [57] again over a narrow range of conditions. Further extensive explorations are required, however, before firm conclusions can be reached.

#### (iv) Examples of future work

Readers will recognize that the results shown earlier, while containing interesting and encouraging pointers, represent just a start on a range of CFD comparisons that will need to be made. As a first step, the computations need to consider a complete rotor rather than just the section between one disc and the next. This will enable the exploration of end effects and the possible variation with height of the wind velocity to be examined as well as potential interference effects between the rotors (if, as in figure 9, a multi-rotor vessel is chosen). The effect of heeling of the ship (even by only around 2–3^°^) on the rotor’s aerodynamic performance also needs to be examined. As a final example of issues requiring examination, we note the possible effects of the top disc of the rotor on the behaviour of the salt-water spray discharge. One may well wish to cause the spray to spread as quickly as possible to minimize the risk of droplet collisions (which would create a larger than optimal size of droplets). It is known that imparting swirl to the spray will do that. However, that will lead to a reduction in the droplets’ vertical velocity, which, on its own, may reduce the proportion of salt particles reaching the cloud base. Such competing effects and their consequences need to be considered in the next phases of this research.

## 6. A limited-area experiment to explore the fundamental processes involved in marine cloud brightening

Before any geoengineering scheme based on SRM could be implemented, it first must fulfil the following criteria: (i) it can deliver the desired agent by which solar radiation will be scattered to space (sulphate particles in the case of a stratospheric aerosol and increases in sea spray aerosol in the case of cloud brightening); (ii) it can deliver the desired radiative response; and (iii) the undesirable climatic responses to geoengineering perturbations are minimal; certainly, they should be no worse than those associated with changes induced in the climate system from the inadvertent human activity that geoengineering is aiming to mitigate. The last of these cannot be tested by experiment for any of the SRM methods without full implementation lasting multiple years and carries a risk of substantial negative impacts. This was argued by Robock *et al.* [58], who focused upon geoengineering through stratospheric sulphur injection, currently considered to be one of the most feasible schemes [59]. Robock *et al.* [58] further argued that it is impossible to fully field test geoengineering schemes without significant modification to the climate system owing to non-local climate responses.

While we agree that large-scale field testing of any geoengineering scheme is indeed inseparable from deployment, small-scale field testing will be necessary to make significant progress in understanding the feasibility of geoengineering schemes [60]. An attractive aspect of MCB in terms of field testing is that, because aerosol particles in the MBL are extremely short-lived (typically a few days) compared with their stratospheric counterparts (1–2 years), perturbations to the radiative budget from MCB are inherently localized. This is not the case with stratospheric sulphur injection. This essentially means that it is possible to conduct a useful test of MCB (with minimal climate impacts) over a limited area that includes testing of TOA radiative responses in addition to the testing of injection methodologies and dispersion, etc. This is in contrast to stratospheric sulphur geoengineering, in which case, as Robock *et al.* [58] correctly argued, it would be extremely difficult to measure either an effect on the Earth’s radiation budget or maintenance of the aerosol in the stratosphere using only a small number of injections that might constitute a field test.

The stratospheric sulphur injection scheme has so far been considered one of the most viable schemes, not least because previous volcanic eruptions such as Pinatubo in 1991 have provided significant data against which model predictions of the radiative effects of sulphate particles in the stratosphere can be tested and validated [61,62]. Unlike stratospheric aerosols, many of the basic processes linking tropospheric aerosols, clouds, precipitation and radiation underpinning the cloud-brightening scheme are rather poorly understood [15]. Given that the influence of human activity on such processes has been proposed to make a substantial contribution to the radiative balance [63,64], it is imperative that basic knowledge of aerosol–cloud interactions is improved substantially, regardless of the viability of cloud brightening as a geoengineering scheme.

Inadvertent human-induced changes to regional aerosol particle burdens have been used to investigate these processes in regions of stratocumulus in the past [65,66], though large natural variability and co-dependency of processes has to date limited progress towards full understanding. Also, emissions from the stacks of ships have been used to study aerosol–cloud interactions [67], but single plumes of this type can provide only limited information as plumes are narrow and entrainment and mixing are often dominant. A limited-area field experiment that provides a substantial and detectable perturbation above the background on spatial scales that are detectable from space could therefore offer a unique way to probe aerosol–cloud–precipitation interactions, and their influence on radiation. It would also enable new knowledge on aerosol influences on climate to be gained.

An analogy can be drawn between improving knowledge of aerosol–cloud interactions through a limited-area perturbation experiment and previous experiments conducted to investigate the control of micronutrients (notably iron) on the drawdown of carbon by marine biological systems. A number of experiments have been conducted, which have deliberately added iron to the ocean to improve knowledge of ocean biological carbon cycling. These have substantially improved knowledge of nutrient limitation on oceanic primary production, its subsequent control on plankton communities and how this impacts on cycling of carbon and nitrogen in the world’s oceans [68]. Further fertilization experiments to develop knowledge of the fundamental processes are seen as crucial to furthering the understanding of the Earth system and are critical before any consideration is given to large-scale deliberate attempts at carbon sequestration by such means [69]. A major concern is that larger scale experiments may have significant impacts on ocean ecosystems. A key point is that a limited-area field experiment to study aerosol–cloud interactions using artificially generated aerosol from sea spray can be carried out without any climatically damaging effects as the lifetime of atmospheric aerosol in the MBL is of the order of a few days at most. Such experiments therefore offer a valuable contribution to climate science and should not be viewed as solely a means of validating the cloud-brightening scheme.

Here, we present an initial framework for the testing and implementation of such experiments. We propose a set of field tests to critically assess the efficacy of MCB over a limited area. The tests are *de minimus* with respect to their climate effects, as we shall discuss later. The tests involve three phases, with increasing logistical complexity, each of which is designed to test one or more important components of the cloud-brightening scheme. Each involves the introduction and monitoring of controlled aerosol perturbations from one or more ship-based seeding platforms up to a limited area of approximately 100×100 km^2^. A suite of observational platforms of increasing number and complexity, including aircraft, ships and satellites, will be required to observe the aerosol plume and in the latter experiments the cloud and albedo responses to the aerosol perturbations. These include the necessary cloud physical and chemical processes that determine the efficacy of the cloud-brightening scheme and are central to the broader questions of aerosol–cloud interactions. Multi-scale modelling work will be carried out to simulate/predict the cloud responses. The modelling work will be used to drive quantitative hypothesis testing for the field tests, and will be used to test our understanding of, and ability to simulate, aerosol–cloud interactions on the regional scale.

The proposed experiments are on a similar scale and complexity to those being routinely conducted by the international research community through inter-agency cooperation.^2^ Such integrated inter-agency collaboration will be necessary to deliver a limited-area field test of aerosol–cloud–precipitation interactions generated by a sea spray generation system. The field testing would need to be conducted in an open and objective manner, in accordance with the Oxford principles of geoengineering governance [70]. Further, they should be sufficiently small to not have inadvertent climate impacts, and certainly within an internationally agreed ‘allowed zone’ [71] to be determined through consultations between high-level international scientific organizations and other potential stakeholders. We return to this point later.

The recommended approach is to test any sea spray generation method, its effect on the cloud system and subsequent radiative impacts through a series of field trials of increasing complexity and expense. The first phase is to establish the ability of a full-size spray generation system to deliver sea spray particles of the correct size and number in such a way that they become mixed throughout the depth of the boundary layer. The second phase would be to use a single system to investigate cloud responses. Because it involves a different suite of cloud measurement instruments and a more complex array of platforms, phase 2 would commence only after the spray system and dispersion has been tested (requirements for success are provided below), and we anticipate that several attempts at phase 1 will be required to refine the spray generation methodology. The third phase would be to conduct a multi-source limited-area experiment at the 100×100 km^2^ scale. Such a strategy assesses viability at each stage without incurring unnecessary risk or expense.

### (a) Field phase 1: injection and dispersion of particles

Technology to create the large number of small particles that can act as CCN on which cloud droplets will form will need to be field tested to ensure that the delivery mechanism (here termed injection) can deliver particles in sufficient quantity and of the appropriate size into the MBL, and to study the dispersion of the aerosols throughout the MBL.

The seeding technology should be deployed on a ship or barge platform in a marine region favourable for MCB. Only a single aircraft fitted with state-of-the art aerosol measurement technology would need to be deployed to sample the aerosol plume as a function of the distance downwind of the injection source.

This study does not need to be carried out in the remote ocean boundary layer and could be located near to the coast for convenience during the early stages of testing of the engineering system. Studies of diesel-burning commercial shipping indicate that a single source will generate a plume that is typically 10 km wide at a distance of 100 km downwind [72]. The aircraft would be used to examine the physical and chemical characteristics of particles (size distribution, chemical composition and cloud-forming properties) close to the injection source and to examine how these particles disperse in the boundary layer with distance downwind. Tracer technology should be used to unequivocally identify the plume and hence record if concentrations of sea salt are undetectable from the background. No attempt should be made in phase 1 to study the cloud responses to the aerosol plume. To do so would significantly increase the complexity and cost of the experiment and would represent a risk were any given generation scheme to fail to deliver the required perturbation. Modelling activities in this phase should focus on the examination of the processes associated with the formation of particles and their modification in the stack, and on aspects of the dispersion and mixing of aerosols throughout the boundary layer downwind of the source.

Phase 1 would be considered successful if the aerosol concentrations measured approximately 100 km downwind of the spray are sufficient to result in significant increase in aerosol concentration and enhanced CCN burden. From the parcel modelling studies described in §4, there is a need to increase the cloud droplet concentration from background values of perhaps 50–100 cm^−3^ to values of 200–400 cm^−3^, which previous estimates suggest requires a sprayer source rate of approximately from 10^15^ to 10^16^ particles per second [4,5]. Particles with salt masses greater than approximately 10^−16^ kg are optimal for seeding (see §4). Wang *et al.* [16] used a single source generating 10^16^ particles to seed a domain of 60×120 km^2^ and obtained significant albedo enhancements in simulations of non-precipitating stratocumulus. Measurement of CCN concentrations within an approximately 10 km wide plume that are consistently several hundred cm^−3^ would constitute a successful phase 1 trial.

### (b) Field phase 2: single source cloud responses

**Figure 13.**
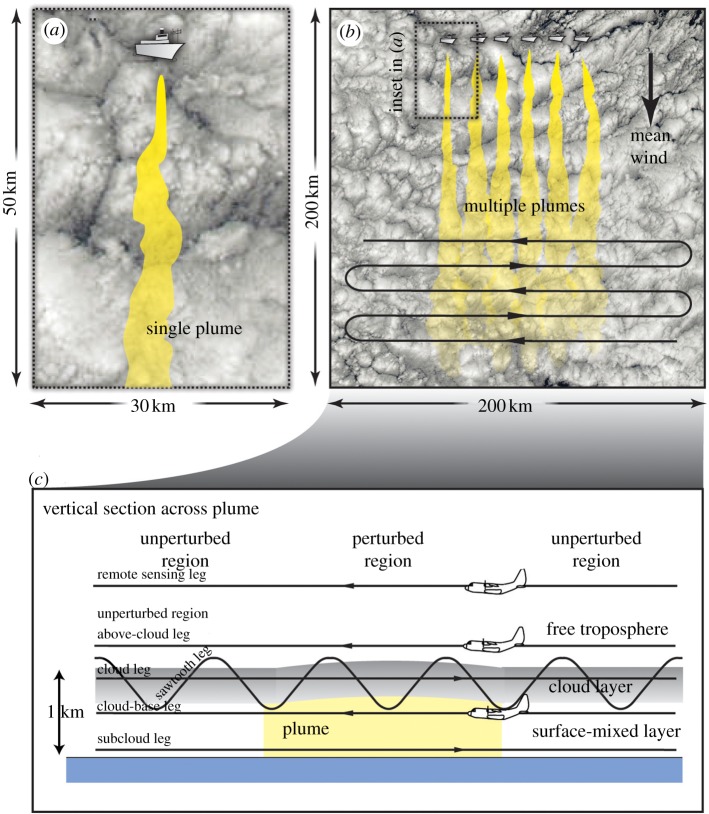
Schematic of the proposed phase 2 and 3 field testing to evaluate the cloud responses to (*a*) a single-seeded plume; (*b*,*c*) multiple-seeded plumes. Examination of ship tracks from commercial ships [72] tells us that the plumes spread quasi-linearly with time at a rate of approximately 2 km h^−1^ [78], which for typical wind speeds of 5–10 m s^−1^ is a width of approximately 6–12 km at a distance of 100 km downwind of the source (*a*). For phase 3 testing, 5–10 ships (six shown in the example here) would be spaced approximately 10 km apart to generate a single plume 50–100 km wide at a distance of 100 km downwind (*b*). This broad plume and its surrounding unperturbed cloud would be sampled in the crosswind direction by stacked aircraft as discussed in the text (*c*). (Online version in colour.)

Once the injection and dispersion technology has been tested and the aerosol plume characterized, the next stage is to examine the cloud responses to a single injection source. The cloud response to a single source will take the form of a ship track (albeit a deliberately produced one). Ship tracks are commonly observed features in regions of marine stratocumulus [72–75] and are associated with small particles emitted from large, commercial, diesel-burning ships [76]. There are existing field observations of ship tracks (e.g. the Monterey area ship track experiment in 1994; [77]). Figure 13 shows a schematic of the scale of such a plume. Ship tracks from commercial ships are typically 300 km in length and approximately 10 km wide a few hours downwind of the emitting ship [77].

Measurement both of the aerosol characteristics below the cloud and of the cloud physical processes should be made with multiple aircraft platforms. The goal would be to test the sensitivity of the cloud microphysical properties to the aerosol perturbations in the plume and to contrast these with the surrounding unseeded clouds under a range of conditions. Once again, releasing a tracer from the spray generation system would provide a useful identification of the plume position. Combinations of volatile organic carbon compounds with varying chemical lifetimes can be used to not only identify the plume but also determine its photochemical age, and these can be identified online using modern mass spectrometric or online chromatographic methods.

Success in phase 2 tests would require ship tracks that are readily detectable both as increased cloud droplet concentrations and reduced droplet sizes from the aircraft flying in the cloud layers, and from space using visible and near infrared satellite imagery. Particular emphasis would be placed upon trying to quantify and understand the extent to which the liquid water contents in the seeded clouds remain unchanged in the seeded area, or whether they decrease as some satellite measurements appear to indicate [79].

Modelling work would be conducted with both process-scale cloud models (see §§3 and 4) and climate models, to test the observed cloud microphysical and macrophysical responses. These modelling studies would also be used to quantitatively predict the outcome of introducing multiple injection sources, which is the key task in phase 3 testing.

### (c) Field phase 3: multiple source limited-area experiment

In the third phase of the proposed field trials, multiple (between 5 and 10) injection sources (figure 13) would be used to create a line (of order 100 km long) of injection sources approximately perpendicular to the mean wind. The plumes from these sources would disperse and would create a single broad perturbed area extending from the source line several hundred kilometres or more downwind. At such scales, the changes in the cloud-filled boundary layer as a result of the doping by particles should be detectable from space if the radiative impact is significant.

Multiple observational platforms should be used to study: (i) the aerosol physical and chemical properties below the cloud inside and outside the seeded area; (ii) the cloud microphysical, structural and dynamical response; and (iii) the cloud albedo response. Measurements should be made at different distances downwind of the source line. Aircraft flights at stacked levels below cloud, in cloud and above cloud would be complemented by a research ship that would continuously sample the air at a variety of distances downwind of the source line (figure 13*c*). Control experiments could be performed in two ways: (i) *spatial control* would involve contrasting the seeded area with the surrounding region and (ii) *temporal control* would involve temporal modulation of the source strength, perhaps with a 6 hour on–off frequency. The required duration of the entire field test would probably be one to two months, which would permit perhaps 15–20 aircraft case studies under different meteorological conditions and under different background aerosol regimes. Sufficient temporal control modulation would be available on these time scales to provide adequate constraints for model studies.

The albedo response to the aerosol perturbations would be quantitatively determined using a combination of airborne and satellite remote sensing. One of the research aircraft would be dedicated to remote-sensing measurement of the shortwave and longwave radiation field above the clouds. Perturbations of several tens of W m^−2^ are expected, which should be readily detectable compared with the background control cloud either side of the perturbed region. Process scale and climate modelling should be performed to quantitatively test the MCB hypothesis. This should involve studies designed to calculate the expected magnitude of the albedo perturbations as a function of the seeding strength and meteorological conditions and to compare these with the observations. In addition, the effects of seeding on the cloud dynamical fields and on the precipitation they produce both need to be determined using state-of-the-art cloud physics and aircraft radar/LIDAR remote-sensing measurements. These would be used to examine the effects on precipitation as a function of distance downstream of the source. On the basis of ship track studies [72], the radiative effects of the seeding are likely to become indistinguishable from the background cloud within 200–300 km downwind of the source, which suggests that precipitation impacts are also likely to be confined to within this distance from the source.

Passive, inert tracers would also be released from the ships (as is the case in all other phases of the work) to provide a control to examine how the particle size distributions are modified with distance downstream. Relative falls in the concentrations of particles with respect to the tracers would provide unprecedented information about the lifetime of the spray particles in the MBL. These would also have the benefit of providing unique data on the cloud top entrainment processes upon which ship track responses are now thought to be critically dependent [27,28].

Phase 3 success would comprise changes in reflected solar radiation within the seeded area of several tens of W m^−2^, because this kind of change would be required in regions of marine stratocumulus to offset the radiative forcing due to anthropogenic greenhouse gases. An experiment that did not produce brightening in excess of 10 W m^−2^, given increases in aerosol particles that parcel models demonstrate to be sufficient (§4), would be considered unacceptable, because it is almost inconceivable that this could be made to generate brightening of sufficient magnitude even if scaled up to all marine areas. Results that deliver changes greatly in excess of 100 W m^−2^ would be termed successful because in this case the seeding could be scaled back to produce results of the desired magnitude.

### (d) Location

Because MCB is aimed at brightening marine stratocumulus clouds, it would be natural to pursue an experiment in a region that frequently experiences this type of cloud. Further, because the increase in albedo owing to the addition of a quantity of additional CCN is greatest for regions with low background concentrations [80,81], it would make most sense to conduct our proposed MCB test in one of the quasi-permanent sheets of marine stratocumulus, and sufficiently far from continental pollution influences that the radiative susceptibility is high. The northeastern or southeastern subtropical Pacific Ocean would be excellent choices. In addition, the field tests should be conducted sufficiently far upstream of landmasses so that the aerosol loading is able to return to normal background values by the time the advected air masses reach landfall. The larger Pacific Ocean basin would perhaps be more appropriate in this regard, although with typical aerosol lifetimes in the boundary layer of 1–2 days, either the Atlantic or the Pacific Basins would be suitable without due concern.

### (e) Climatic impacts of marine cloud-brightening field testing

To ensure that the climatic effects of our proposed MCB field experiments are negligible, we argue here that they will satisfy two important and stringent criteria:

- The experiments do not cause detectable climatic responses inside or outside the region defined to be part of the experiment.
- The experiment does not cause damage to the ecosystem.

We argue that detectable climatic responses of repeated MCB would require the SST to be lowered by several tenths of a Kelvin over the 100×100 km^2^ area. Phase 3 has the greatest potential impact. The proposed experiment would be conducted over a period of perhaps two months (to ensure a sufficient number of flights). To allow sufficient time for aerosols to disperse and to impact the low clouds and the radiation field, for each of the 15–20 flights, the spray generation system would need to be operated for perhaps a 6–12 hour period. The mean perturbations to the TOA solar radiation needed in regions of marine stratocumulus to produce a sufficient global response to counter anthropogenic greenhouse gas warming is of the order of 20–40 W m^−2^ (see fig. 3 in Latham *et al.* [5]). Because solar radiation is zero at night, daytime mean values of 40–80 W m^−2^ are needed. Atmospheric absorption changes and longwave perturbations are expected to be small, and so, in a two-month period, the mean perturbation to the surface net radiation budget from 20 instances lasting 12 hours during daylight would be approximately 10 W m^−2^. For an oceanic mixed layer depth of 50 m, which is typical in regions of subtropical marine stratocumulus [82], a net radiative perturbation of this magnitude would lead to a cooling of the SST of approximately 0.25 K over the 100×100 km^2^ experimental domain. To assess the detectability of such a systematic cooling, we can compare this number with fluctuations in SST typically associated with similarly sized ocean mesoscale eddies that are comparable in magnitude [83]. Large mesoscale eddies are common over the subtropical oceans where MCB experiments would be likely to take place [84]. It would therefore be difficult to detect impacts of MCB experiments on SST against the backdrop of natural oceanographic variability. In addition, it is difficult to argue that such a small perturbation to the SST over a region on the scale of a mesoscale ocean eddy can produce a significant climate impact. Nevertheless, it would be responsible for conducting high-resolution regional climate model simulations prior to conducting the field trials in order to provide assurance that climatic responses to such perturbations would indeed be negligible. Evidence of detectable remote impacts from the simulations would be sufficient to prevent field testing.

Further, it is difficult to conceive of significant ecosystem impacts of the experiment. The SST changes are small, and because the salt used to generate the aerosol particles originates and is returned to the ocean surface fairly locally, salinity and other nutrients are not significantly impacted. Changes in the level of illumination at the sea surface are relatively small (several times smaller than they would be for full-scale deployment), but further work will be needed to understand fully the potential ecosystem impacts [85].

## 7. Discussion

The multi-faceted research described in the preceding sections and conducted by our rather amorphous ‘team’ of scientists and technologists can be summarized as follows.

Several GCM studies ([5,6] and Jones *et al.* [7,8,10]) yield the conclusion that—subject to satisfactory resolution of all of a number of important issues, described earlier—MCB could produce a globally averaged negative forcing of significance. A detailed study by Korhonen *et al.* [9] predicts appreciably lower forcing, and this study outlines possible reasons for this disparity. Our GCM modelling confirms the results of studies by Jones *et al.* [7], which show that MCB could produce unacceptable rainfall reduction in the Amazonian region of South America. However, Jones *et al.* [8] show that this reduction could be circumvented by not seeding in a particular area. This study also provides some new results regarding the influence of MCB on sea-ice thickness. Our high-resolution cloud modelling underlines earlier work on the complexities of marine stratocumulus clouds, and shows how the negative forcing produced by cloud seeding is sensitive to both cloud characteristics and seeding strategy. Cloud parcel modelling provides estimates of the ranges of sprayed sea water droplet sizes and salt masses that would be effective for cloud droplet activation, as a function of cloud characteristics. This information is required for the development of the spray generators/disseminators for cloud seeding. Current work on one possible spray system—electrohydrodynamic spray fabrication—is described, while an alternative system involving microfabrication lithography was presented in Salter *et al.* [4]. More testing of both techniques is required. This earlier (2008) work also provided detailed information on an updated version of unmanned, satellite-guided, wind-powered Flettner-rotor vessels, which could be the vehicles from which the spray droplets would be disseminated, if MCB was ever to be deployed. Here, we present CFD studies of possible instabilities in Flettner rotors. Finally, we summarize current thinking regarding a possible three-stage quantitative field study of MCB, designed to determine whether cloud seeding with sea water aerosol can increase cloud albedo, and, if so, to what degree and under what circumstances. This study—which we envisage would be performed on a spatial scale of about 100×100 km^2^, and is not designed to examine possible effects on climate—should also yield useful fundamental information on these climatologically important clouds. As already stated, deployment of MCB should never occur unless approved by the relevant international authority, and shown, via intensive modelling studies, to have no likelihood of significant adverse consequences.

It is unclear whether deployment of the MCB geoengineering technique would be warranted, even if the climate-change problem reached such a drastic stage that some form of intervention was deemed to be required. GCM modelling by three independent groups, using three different models [6,7,10], indicates that, if it functioned as assumed in the modelling, it could—roughly—stabilize the Earth’s average surface temperature and maintain current levels of polar sea-ice cover at approximately current values for some decades, at least up to the carbon dioxide-doubling point, where the required negative forcing for full compensation is approximately −3.7 W m^−2^. The computations of Korhonen *et al.* [9], discussed in §2, yield significantly lower values of negative forcing. This disparity may result from the usage of appreciably different values of natural (no-seeding) CDNCs, *N*_0_ (see §§1 and 2) or possibly the vertical velocity field values used in their simulations were too small. In practice, it may be possible to reconcile these disparate results by increasing the dissemination rate of sea water aerosol assumed in the Korhonen study—which we believe would be feasible technologically. However, as discussed in §4, marine stratocumulus clouds are much more complex than has been implicitly assumed in this modelling, and considerably more fundamental research into these clouds is required before we can establish whether our assumptions are justified to an acceptable degree. Also, we have not yet established—for all situations of interest—quantitative values for the fraction of spray droplets generated at or near the ocean surface that enter the bases of the clouds above. Nor have we succeeded to date in developing a sea water spray-production system that meets our requirements as to droplet size and spray rate. Finally, we have not yet thoroughly examined the (possibly adverse) ramifications of deployment of the technique. No case for deployment would exist unless it was established that all such deleterious effects of significance could be remedied. We need constantly to keep in mind that, while some areas may benefit from MCB geoengineering, there may well be regions where the response is significantly detrimental. If so, and if this situation could not be corrected, deployment of MCB would not be justified.

Two advantages of MCB, in principle, are that (i) the sprays could be switched off immediately, with essentially all of the sea water droplets returning to the ocean within a few days, and (ii) because, for some decades, not all suitable clouds would need to be seeded in order to produce sufficient negative forcing to balance the carbon dioxide increase, there exists, in principle, flexibility to confine the seeding to selected cloudy areas which produce no adverse consequences or reduce them to acceptable levels. However, item (i) just mentioned above could prove to be a serious disadvantage, because MCB is more vulnerable to attack than other leading SRM techniques, the spray vessels being located around the oceans. If all or some significant fraction of the fully deployed vessels were destroyed or otherwise rendered unworkable a rapid rise in temperature would be initiated, with concomitant changes in weather patterns and other adverse consequences. This would be true whether the vessels were powered by the wind or by burning fossil fuel.

If MCB proves to be viable, and deployment of an SRM scheme necessary, optimal beneficial cooling might be produced if it was used in concert with another possibly viable technique (e.g. stratospheric sulphur seeding [59], or microbubble ocean whitening [86]). In the former case, for example, the primary cooling could be supplied by the stratospheric scheme, with beneficial adjustments being made by MCB, which can function in a more localized manner. It may even prove possible and useful to create localized warming via seeding, to optimize this fine tuning.

Other issues that might be addressed by exploiting the initially localized cooling of oceanic surface waters that we hope would be produced by MCB (and/or the microbubble technique) are coral reef protection and hurricane weakening. In the latter case, it may prove possible to cool oceanic waters in the regions where hurricanes spawn. This would probably require continuous seeding over several months, culminating in the hurricane season. Also, it may prove possible to produce sufficient polar cooling to maintain existing sea-ice cover by seeding specially selected cloudy regions of much smaller total area than considered in our study [6].

Bala *et al.* [10] found that when MCB was used in a carbon dioxide-doubled environment the cooling associated with cloud seeding was a maximum in the two polar regions, compensating roughly for the preferential warming resulting from the additional carbon dioxide. Our own modelling (§2) has produced similar results. A comprehensive series of model inter-comparisons is urgently required in order to optimize and better quantify our understanding and assessment of MCB. We must also conduct a parallel programme of fundamental research into the associated cloud physics and chemistry, aerosol properties and transport, meteorology, etc.

As mentioned earlier, Bala *et al.* [10] also found that, if all suitable clouds were seeded, MCB would cause a decrease in globally averaged rainfall, but a net increase in rainfall over land. They surmised that this latter effect occurred because the cooling produced by MCB set up air circulations that brought moist air from ocean to land.

If satisfactory resolution of all significant problems associated with MCB, identified earlier, were to be achieved, and a need for its deployment was deemed to exist, it would be necessary to make an informed decision as to the type of vessel to be used for spray dissemination. Seeding from aircraft is one possibility. Alternatively, in principle, nuclear-powered vessels could be used. However, Salter *et al.* [4] focused attention on wind-powered, unmanned, satellite-guided Flettner ships, and it was estimated that about 1500 spray vessels, each consuming about 150 kW (derived from the wind), would be required to produce the globally averaged negative forcing of −3.7 W m^−2^ required to balance carbon dioxide doubling. Flettner ships have the advantages of low cost, high manoeuvrability and low carbon footprint. A conventionally, powered ship might consume about 1 MW; so, for both types of vessel, the ratio of the rate of planetary radiative loss to required operational power is very large (in the range from 10^5^ to 10^7^). It follows that considerations of energy efficiency, desirable though that is, should not dictate the selection of type of spray vessel. Latham *et al.* [5] pointed out that the main reason that this ratio is so high for MCB is that Nature provides the energy required for the increase of surface area of newly activated cloud droplets by four or five orders of magnitude as they ascend to cloud top and reflect sunlight.

The earlier mentioned arguments are based on the assumption that current GCM modelling is reasonably accurate. However, if it transpires that estimated albedo change/droplet flux ratio values are seriously inflated because, for example, of significant overestimation of the fraction of disseminated sea water particles that rise into the clouds, this issue would need to be reassessed. Other factors then to consider include the levels of pollution produced by spray vessels and the energy they consume. It is also to be noted that, during the decades leading to carbon dioxide doubling, the amount of negative forcing required of MCB would be correspondingly less, as would the emissions (which would be very low for wind-powered Flettner vessels). Definitive statements on these issues must wait on further research, on all fronts covered in this article.

## Footnotes

1 is the wind velocity past the rotor of diameter *D* and *ν* is the kinematic viscosity of the air.

2 For example, the variation in the American monsoon system (VAMOS) ocean cloud atmosphere land study (VOCALS) was developed to improve understanding of the southeast Pacific coupled ocean–atmosphere–land system on diurnal to inter-annual time scales. A large component of VOCALS centred around a large-scale regional experiment to investigate the interactions between aerosol, cloud and precipitation across a strong pollution gradient in a region dominated by the largest and most persistent stratocumulus cloud sheet on the planet [18]. The field experiment involved the use of five aircraft and two research ships, operating in the region between 70 and 80 W at a longitude of around 20S for a period of around four to six weeks and received multi-agency and multi-national support.
